# Mucosa-Colonizing Microbiota Correlate With Host Autophagy Signaling in Patients With Inflammatory Bowel Disease

**DOI:** 10.3389/fmicb.2022.875238

**Published:** 2022-05-26

**Authors:** Wenxue Wang, Zhongjian Liu, Wei Yue, Ling Zhu, Huijie Zhong, Chao Yang, Tian He, Ping Wan, Jiawei Geng

**Affiliations:** ^1^Department of Infectious Disease and Hepatic Disease, First People’s Hospital of Yunnan Province, Affiliated Hospital of Kunming University of Science and Technology, Kunming, China; ^2^School of Medicine, Kunming University of Science and Technology, Kunming, China; ^3^Institute of Basic and Clinical Medicine, First People’s Hospital of Yunnan Province, Affiliated Hospital of Kunming University of Science and Technology, Kunming, China; ^4^Department of Gastroenterology, First People’s Hospital of Yunnan Province, Affiliated Hospital of Kunming University of Science and Technology, Kunming, China; ^5^Faculty of Life Science and Technology, Kunming University of Science and Technology, Kunming, China

**Keywords:** autophagy, ER stress, bile, microbiome, transcriptome, inflammatory bowel disease

## Abstract

Both bacteria and autophagy are implicated in inflammatory bowel disease (IBD) pathogenesis. However, how bacteria crosstalk with autophagy signaling remains largely known, especially in intestinal mucosa. This study aimed to profile the internal complex autophagy signaling cascade and their external correlation with these bacteria, and consequently provide a systematic and precise target for future IBD diagnosis and therapy. We found the Ulcerative colitis (UC) patients exhibited more severe dysbiosis than the Crohn’s disease (CD) patients, as represented by alpha diversity, community phenotypes, and functional annotation compared with the control population. Meanwhile, CD patients showed greater transcriptional signaling activities of autophagy, endoplasmic reticulum (ER) stress, and bile acid production. Dominant bacteria (e.g., *Rhodococcus*, *Escherichia*, *Shigella*, and *Enterococcus*) were positively correlated and low-abundance bacteria (e.g., *Bacillus*, *Acidovorax*, *Acinetobacter*, and *Stenotrophomonas*) were negatively correlated with the autophagy signaling cascade (184 autophagy genes, 52 ER stress genes, and 22 bile acid production genes). Our observations suggested UC patients showed temporary and widespread microbiota turbulence and CD patients showed processive and local autophagy activity during IBD progression. Intestinal mucosa-colonizing bacteria were correlated with the bile/ER stress/autophagy signaling axis in IBD pathogenesis.

## Introduction

Intestinal microbes and their metabolic products play key roles in human diseases, especially inflammatory bowel disease (IBD). Meanwhile, both adherent-invasive and diffusely adherent bacteria stick to the intestinal mucosa and induce host immune cell activity and are linked to Crohn’s disease (CD) and ulcerative colitis (UC), respectively (Mirsepasi-Lauridsen et al., 2019). Notably, mucosa samples can directly reflect bacteria-host interactions during IBD progression. However, few multi-omics studies have investigated these interactions using mucosal biopsies. Furthermore, both the classification and mechanistic dissection of pathways involved in IBD remain challenging due to the complex correlative and interactive networks between host genetics and microbes (Plichta et al., 2019). Therefore, mucosal biopsy specimens are required to further explore these networks between host genetic factors and microbiota.

Genome-wide association studies have indicated that autophagy is an important mechanistic dissection of IBD pathogenesis, especially that of CD (Nguyen et al., 2013). Autophagy not only decreases intestinal epithelial permeability by inducing lysosomal degradation of tight junction proteins (Nighot et al., 2015), but also modulates programmed cell death in the intestinal epithelium (Matsuzawa-Ishimoto et al., 2017). In addition, autophagy is responsible for the elimination of intercellular bacteria from endoplasmic reticulum (ER) function defect-induced intestinal barrier leakage. For instance, autophagy can be induced by Autophagy related 16 like 1 (ATG16L1)-Transmembrane protein 59 (TMEM59) interactions in response to bacterial infection (Boada-Romero et al., 2016). Furthermore, activation of the bile acid receptor can strongly suppress the induction of autophagy (Lee et al., 2014). Thus, the profiles of mucosal bacteria-correlated autophagy gene networks and their interactions with ER stress and bile production signaling should be clarified in IBD patients.

Here, we collected intestinal mucosal biopsies from control population and IBD patients for dual-omics analysis of mucosal transcriptome and mucosa-colonizing bacterial diversity. Analysis focused on the autophagy signaling cascade and their complex correlations with intestinal mucosa-colonizing bacteria in IBD patients and control population. Combined with previous studies, our analysis suggested that mucosal bacteria regulated both ER stress and bile acid production and consequent autophagic activity, finally acting on host IBD progression. We systematically profiled the mucosal bacteria-autophagy correlation network in IBD patients, especially in those with CD, and provided a reliable analysis model of microbe-correlated intestinal diseases.

## Materials and Methods

### Study Design

The control populations and IBD patients were recruited from the First People’s Hospital of Yunnan Province, China, from October 2017 to December 2019. Each participant provided information on age, occupation, and smoking and alcohol drinking history. The control population had no history of digestive tract disease or serious medical illnesses. Each IBD patient was diagnosed with UC or CD and had received no IBD therapy or antibiotics (within 3 months). All participants provided signed informed consents and completed a questionnaire regarding their age, sex, occupation, and antibiotic use, with family assistance if necessary.

#### Diagnosis of IBD

We used clinical feature inquiry, laboratory examination, and endoscopy and biopsy histopathological analysis to diagnose UC or CD according to clinical practice guidelines (Gomollón et al., 2017; Magro et al., 2017; Inflammatory Bowel Disease Group, Chinese Society of Gastroenterology, Chinese Medical Association, 2021). Firstly, outpatients with enteric symptoms of abdominal pain, diarrhea, blood in stools, and loose stools for more than 6 weeks, and/or parenteral manifestations, such as fatty liver and cholecystitis, were suggested for laboratory examination, which included a full blood count, electrolyte, liver function, and inflammatory marker [C-reactive protein (CRP)] tests. Secondly, when clinical features and laboratory tests suggested further investigation, an endoscopy (colon and/or intestinal endoscopy) was performed to obtain evidence and collect mucosal biopsies for histopathological analysis. Thirdly, histopathological investigations were performed to provide reliable information on mucosal architecture, lamina propria cellularity, neutrophil granulocyte infiltration, and epithelial abnormality. Finally, UC or CD was diagnosed when comprehensive analysis of clinical features, laboratory examinations, endoscopy, and histopathology gave positive responses.

#### Exclusion Criteria and Control Population Selection

To eliminate the possible effects of other intestinal diseases on UC and CD diagnosis, we carefully discriminated infectious enteritis, *Clostridium difficile* infection, intestinal tuberculosis, Behçet syndrome, amebiasis, schistosomiasis, and other intestinal diseases from IBD. Those participants without IBD or other digestive tract diseases and without serious diseases of other tissues and organs were chosen as the control population group. A total of 138 participants were initially recruited, 38 of which were excluded according to exclusion criteria (i.e., UC and/or CD and antibiotic use within the last 3 months). Finally, 25 control population, 26 CD patients, and 51 UC patients were enrolled in the study.

#### Sample Collection

The IBD patients and control population were first given an intravenous injection of propofol and etomidate, and then an enteroscopy was performed for mucosal sampling. All samples were collected from October 2017 to December 2019. Upon collection, the mucosal samples were immediately placed on ice and frozen at −80°C within 1 h for microbiome and transcriptome analyses.

### Bacterial DNA Isolation and 16S rRNA Gene Sequencing

Microbial community genomic DNA was extracted from intestinal mucosa using a QIAamp DNA Mini Kit (Qiagen, Germany) according to the manufacturer’s instructions. The DNA extract was checked on 1% agarose gel, and DNA concentration and purity were determined using a NanoDrop 2000 UV–vis spectrophotometer (Thermo Scientific, Wilmington, United States). The hypervariable V3–V4 region of the bacterial 16S rRNA gene was amplified with primer pairs 338F (5′-ACTCCTACGGGAGGCAGCAG-3′) and 806R (5′-GGACTACHVGGGTWTCTAAT-3′) using an ABI GeneAmp® 9700 PCR thermocycler (ABI, CA, United States). PCR amplification of the 16S rRNA gene was performed as follows: initial denaturation at 95°C for 3 min, followed by 27 cycles of denaturing at 95°C for 30 s, annealing at 55°C for 30 s, extension at 72°C for 45 s, single extension at 72°C for 10 min, and end at 4°C. The PCR mixture contained 5× TransStart FastPfu buffer 4 μl, 2.5 mM dNTPs 2 μl, forward primer (5 μM) 0.8 μl, reverse primer (5 μM) 0.8 μl, TransStart FastPfu DNA Polymerase 0.4 μl, template DNA 10 ng, and finally ddH_2_O up to 20 μl. PCR was performed in triplicate. The PCR products were extracted from 2% agarose gel and purified using an AxyPrep DNA Gel Extraction Kit (Axygen Biosciences, Union City, CA, United States) according to the manufacturer’s instructions and quantified using a Quantus™ Fluorometer (Promega, United States).

Purified amplicons were pooled in equimolar concentrations and paired-end sequenced on the Illumina MiSeq PE300/NovaSeq PE250 platforms (Illumina, San Diego, United States) using standard protocols.

The raw 16S rRNA gene sequencing reads were demultiplexed, quality-filtered using fastp v0.20.0 (Chen et al., 2018), and merged using FLASH v1.2.7 (Magoč and Salzberg, 2011) with the following criteria: (i) 300-bp reads were truncated at any site receiving an average quality score of <20 over a 50-bp sliding window, with truncated reads shorter than 50 bp and reads containing ambiguous characters discarded; (ii) only overlapping sequences longer than 10 bp were assembled according to their overlapping sequence. The maximum mismatch ratio of the overlapping region was 0.2. Reads that could not be assembled were discarded; and (iii) Samples were distinguished according to the barcode and primers, and the sequence direction was adjusted, exact barcode matching, two nucleotide mismatches in primer matching.

Operational taxonomic units (OTUs) with 97% similarity cutoff (Stackebrandt and Goebel, 1994; Edgar, 2013) were clustered using UPARSE v7.1, and chimeric sequences were identified and removed. The taxonomy of each OTU representative sequence was analyzed using RDP Classifier v2.2 (Wang et al., 2007) against the 16S rRNA database (Silva v132) with a confidence threshold of 0.7. To remove possible contamination, we sequenced three samples of pure water as a negative control with the same procedures, including DNA extraction, PCR amplification, cDNA library construction, and final sequencing. Based on the negative control sequencing results, the same DNA sequence count detected in the negative control was extracted from all control and IBD samples. To confirm the reliability of 16S rRNA sequencing data, we also performed real-time PCR and found the data are replicable.

### Bioinformatics Analysis of 16S rRNA Gene Sequencing Data

#### Alpha Diversity Analysis

Community richness (Sobs) and diversity (Shannon) indices were used to estimate *α* diversity and examined using the Welch’s *t*-test. Relative abundance of the top 47 genera was displayed using a histogram.

#### Community Hierarchical Clustering

Community clustering heatmap was conducted to show community variation at the genus level (top 47). Multiple sample comparisons were performed using Tukey–Kramer one-way ANOVA with false-discovery rate (FDR) correction and 95% confidence. Significance was assumed for adjusted values of *p* ≤ 0.05 (R software, vegan package).

#### Analysis of Abundance Differences

Significant differences between control population and IBD patients were determined using the Kruskal-Wallis H test at genus level. The FDR and Tukey–Kramer methods (CI = 0.95) were used for multiple testing correction and *post hoc* tests, respectively.

#### Functional Microbial Composition Analyses

The Functional Annotation of Prokaryotic Taxa (FAPROTAX) database extrapolates functions of cultured prokaryotes to estimate metabolic and other ecologically relevant functions (Louca et al., 2016). We used FAPROTAX to assess how the metabolic activities of intestinal mucosa-colonizing bacteria affect the host signaling network using the Tukey–Kramer method (CI = 0.95).

#### Phenotypic Prediction of Intestinal Mucosa-Colonizing Bacteria

Intestinal mucosa-colonizing bacteria phenotypes were predicted and compared using BugBase (Ward et al., 2017) and the Kruskal-Wallis H test. Briefly, BugBase uses an OTU table as an input file, which is normalized by the predicted 16S copy number. The preprocessed database and BugBase tool then automatically select thresholds to predict bacterial phenotypes.

### RNA Extraction and Sequencing Data Processing

#### RNA Extraction

Total RNA was extracted from tissue using TRIzol® Reagent according to the manufacturer’s instructions (Invitrogen), and genomic DNA was removed using DNase I (Takara). RNA quality was then determined using a 2100 Bioanalyzer (Agilent) and quantified using a ND-2000 spectrophotometer (NanoDrop Technologies). Only high-quality RNA samples (OD260/280 = 1.8–2.2, OD260/230 ≥ 2.0, RIN ≥ 6.5, and 28S:18S ≥ 1.0, >1 μg) were used to construct the sequencing library.

#### Library Preparation and Illumina Hiseq X Ten/NovaSeq 6000 Sequencing

The RNA-seq transcriptome library was prepared using a TruSeq™ RNA Sample Preparation Kit (Illumina, San Diego, CA, United States) with 1 μg of total RNA. In brief, messenger RNA (mRNA) was isolated according to the polyA selection method by oligo(dT) beads and then fragmented using fragmentation buffer. The double-stranded cDNA was synthesized using a SuperScript Double-Stranded cDNA Synthesis Kit (Invitrogen, CA, United States) with random hexamer primers (Illumina). The synthesized cDNA was then subjected to end repair, phosphorylation, and “A” base addition according to the library construction protocols of Illumina. Libraries were size-selected for 300-bp cDNA target fragments on 2% low-range ultra-agarose gel, followed by PCR amplification using Phusion DNA polymerase (NEB) for 15 PCR cycles. After quantification by TBS380, the paired-end RNA-seq library was sequenced using an Illumina HiSeq X Ten/NovaSeq 6000 sequencer (2 × 150-bp read lengths).

#### Read Mapping

The raw paired-end reads were trimmed and quality controlled using SeqPrep^1^ and Sickle^2^ with default parameters. The clean reads were then separately aligned to the reference genome in orientation mode using HISAT2^3^ software (Kim et al., 2015). The mapped reads of each sample were assembled using StringTie^4^ with a reference-based approach (Pertea et al., 2015).

#### Differential Expression Analysis and Functional Enrichment

To identify differentially expressed genes (DEGs) between two samples, the expression level of each transcript was calculated according to the transcripts per million reads (TPM) method. RSEM^5^ was used to quantify gene abundances (Li and Dewey, 2011). Essentially, differential expression analysis was performed using DESeq2 (Love et al., 2014)/EdgeR (Robinson et al., 2010) with Q value ≤ 0.05 and DEGs with |log2FC| > 1 and Q value ≤ 0.05 (DESeq2 or EdgeR)/Q value ≤ 0.001 (DEGseq) deemed significant. In addition, Gene Ontology (GO) and Kyoto Encyclopedia of Genes and Genomes (KEGG) functional enrichment analyses were performed to identify DEGs significantly enriched in GO terms and metabolic pathways (Bonferroni-corrected value of *p* ≤ 0.05) compared to the whole transcriptome background. GO functional enrichment and KEGG pathway analysis were carried out using GOATOOLS^6^ and KOBAS (http://kobas.cbi.pku.edu.cn/home.do; Xie et al., 2011).

#### Alternative Splicing Event Identification

All alternative splicing events that occurred in the samples were identified using the recently released program rMATS (http://rnaseq-mats.sourceforge.net/index.html; Shen et al., 2014). Only isoforms similar with the reference or comprising novel splice junctions were considered; and splicing differences were detected as exon inclusion and exclusion, alternative 5′ and 3′, and intron retention events.

### Transcriptome Analysis

#### Time Series Expression Trend Analysis

STEM is used for clustering, comparing, and visualizing time series gene expression data (Ernst and Bar-Joseph, 2006). The STEM clustering algorithm is a supervised algorithm, that is, clustering is classified into artificially set trends. First, the software simulates *n* of the most representative possible trends according to the preset, and then calculates the correlation coefficient between each gene and the preset trends. Finally, each gene is classified into the trend to which it is most similar. We used STEM (v1.3.11) to explore the gene expression pattern of the healthy controls and CD and UC patients. Analysis explored functional enrichment of genes with a certain expression pattern and predicted the genetic regulatory network of intestinal mucosa.

#### Gene Expression Correlation Analysis

Genes obtained from expression trend analysis and/or related to autophagy, ER stress, and bile acid production were used to calculate Spearman correlation coefficients based on the correlation of gene expression. In the correlation networks, the larger the node, the greater the number of correlations between the expression of the gene and other genes.

#### Protein–Protein Interaction Analysis

We used the STRING database^7^ to perform Protein–Protein Interaction (PPI) network analysis of genes of interest. The interactions corresponding to genes of interest were directly extracted from the database to construct the network. NetworkX (Python) was used to visualize the network of the genes of interest.

#### KEGG Functional Enrichment Analysis

We used R script and Fisher’s precision probability test to perform KEGG pathway enrichment analysis. To control the false positives of enrichment analysis, multiple testing corrections (Benjamini-Hochberg, BH) were carried out. KEGG pathways reaching a corrected *p* value of 0.05 were defined as significantly enriched.

### Ethics Approval

All study protocols and procedures were approved by the Medical Ethics Board of the First People’s Hospital of Yunnan Province (GXBSC-2021001, 2021 updated), China, and were carried out in accordance with all relevant provincial, national, and international guidelines, including the Declaration of Helsinki. Written informed consent was obtained from all participants prior to their inclusion in the study.

### Statistical Analysis

GraphPad Prism v9.0.0 (San Diego, CA, United States) and the R stats package were used to analyze all data (R Core Team, 2013). The Kruskal-Wallis H test with FDR correction (CI = 0.95) for multiple comparisons was conducted to compare abundant bacterial taxa. Spearman rank-order correlation was used to evaluate associations between genus-level bacterial relative abundances and autophagy-related gene expression levels. In all analyses, *p* < 0.05 was considered statistically significant.

## Results

### Characteristics of IBD Patients and Controls

We initially recruited 138 participants, 38 of which were excluded due to antibiotic use within the last 3 months and/or suffering from other serious illnesses. In total, 23 healthy control population, 26 CD patients, and 51 UC patients were enrolled (Table 1). Most IBD patients were male (68 out of 77 subjects) and had no history of smoking. The control population was recruited from outpatients suffering abdominal discomfort, but with no gastrointestinal or other serious illness. For transcriptome analysis, an additional biopsy sample was collected from six control population, five CD patients, and 12 UC patients (Supplementary Table S1) with informed consent.

**Table 1:** Basic characteristics of all IBD patients.

Index	Control (*n* = 23)	CD (*n* = 26)	UC (*n* = 51)
Gender (male: female)	11:12	15:11	28:23
Age (mean ± SD)	47.48 ± 11.14	38.77 ± 9.40	44.86 ± 11.13
Smoking (yes: no)	0:23	9:17	11:40
Alcoholic drinking (yes: no)	11:12	7:19	34:17
Antibiotic use (within 1 month)	No	No	No
Occupation			
Farmer	7	6	8
Factory worker	8	7	11
Office staff	3	3	21
Teacher	0	4	3
Others	5	6	8
Chief complaint			
Abdominal pain	5	15	13
Diarrhea	0	2	16
Blood in stools	18	3	19
Others	0	6	3

### IBD Patients, Especially UC Patients, Show Intestinal Mucosa-Colonizing Microbial Dysbiosis and Corresponding Dysfunction

**Figure 1:**
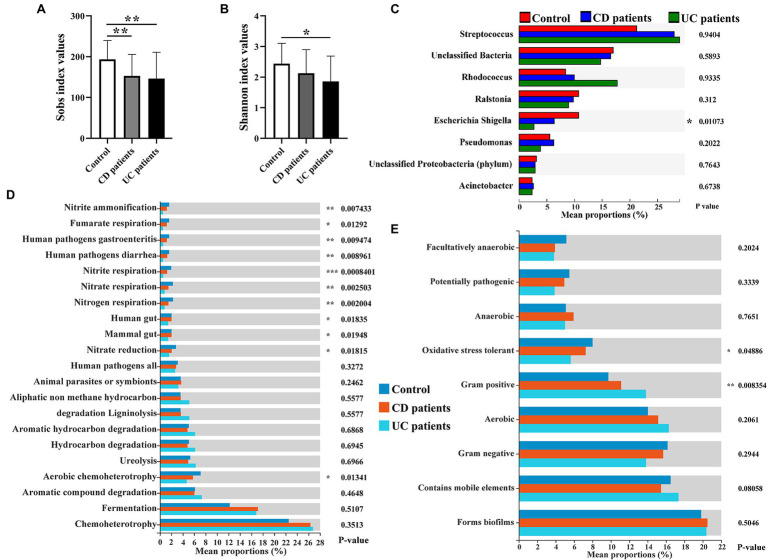
Intestinal mucosa-colonizing bacteria and their potential functional profiles and community phenotypes in control population and inflammatory bowel disease (IBD) patients. Alpha diversity of intestinal mucosa-colonizing microbiota based on Sobs index **(A)** and Shannon index **(B)**. **(C)** Kruskal-Wallis H test compared average composition of intestinal mucosa-colonizing bacteria (top 8) at genus level. Multiple testing correction: false discovery rate (FDR); *Post hoc* test: Tukey–Kramer (CI = 0.95). All bacteria were named to genus level unless otherwise noted in brackets. **(D)** Functional Annotation of Prokaryotic Taxa (FAPROTAX) approach predicted potential functional profiles of intestinal mucosa-colonizing bacteria using Kruskal-Wallis H test. Multiple testing correction: FDR; *Post hoc* test: Tukey–Kramer (CI = 0.95). **(E)** BugBase predicted community phenotypes of intestinal mucosa-colonizing bacteria using Kruskal-Wallis H test. Multiple testing correction: FDR; *Post hoc* test: Tukey–Kramer (CI = 0.95). * 0.01 < *p* ≤ 0.05, **0.001 < *p* ≤ 0.01, and *** *p* ≤ 0.001. Control population: *n* = 23; CD patient: *n* = 26; and UC patient: *n* = 51.

The intestinal microbiota play a key role in IBD progression. IBD patients exhibit clear dysbiosis, mainly represented by a decrease in bacterial diversity (Ott et al., 2004; Manichanh et al., 2006). Mucosal biopsies are advantageous as they can be used to explore the real interactions between dysbiosis and host responses. Here, we collected intestinal mucosal biopsies from control population and IBD patients to investigate the compositional movement and functional variation potential of intestinal mucosa-colonizing bacteria. Consistent with previous study (Ott et al., 2004), we found that alpha diversity (Sobs and Shannon indices) of the intestinal mucosal bacterial community was significantly decreased in IBD patients compared with the control population. Furthermore, UC patients showed poorer alpha diversity than CD patients (Figures 1A,B). The decrease in the Sobs index mainly originated from the reduced abundances of Proteobacteria and Bacteroidetes (Supplementary Figure S1). Correspondingly, FAPROTAX analysis indicated that the metabolic activities of nitrite, nitrate, and fumarate were significantly decreased in IBD patients, especially UC patients (Figure 1D; Supplementary Figure S3). Notably, these metabolic activities had brought focuses because of their key roles in IBD incidence, prevention, and therapy (Dykhuizen et al., 1996; Saijo et al., 2010; Jädert et al., 2014; Casili et al., 2016). Redox of these nitrogenous salts is also associated with IBD pathology (Bourgonje et al., 2020). Based on community phenotype analysis, we also found that IBD patients showed a decrease in oxidative stress tolerance, primarily due to the lower abundances of *Escherichia*, *Shigella*, and *Pseudomonas* (Figure 1E; Supplementary Figures S4A,B). Unexpectedly, pathogen causing diarrhea and gastroenteritis potentials also decreased significantly in IBD patients. Potential pathogens, such as *Rhodococcus*, *Streptococcus*, *Enterococcus*, *Veillonella*, *Ruminococcus torques*, and *Ruminococcus gnavus*, were enriched in the intestinal mucosa of IBD patients (Figure 1C; Supplementary Figure S2), which contributed to high Gram-positive performance, another community phenotype (Supplementary Figures S4C,D).

### Active Intestinal Mucosa Signaling in CD Patients Exceeds That in UC Patients

**Figure 2:**
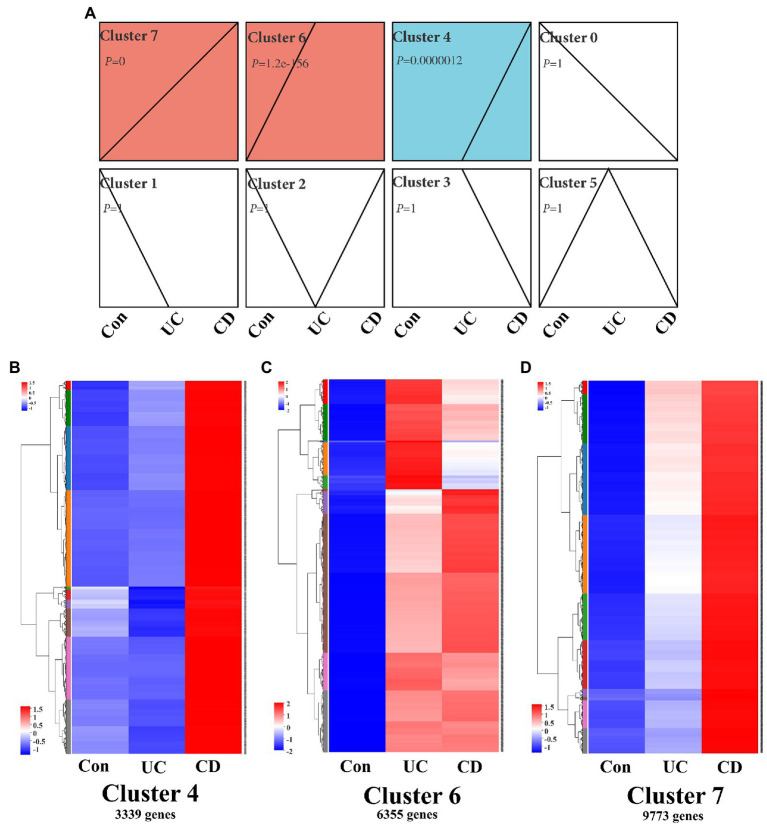
Clustering profile of intestinal mucosa signaling in IBD patients. **(A)** Trend analysis chart showing gene expression profile in intestinal mucosa in IBD patients using a time course analysis. Chart shows all active genes were divided into eight clusters, three of which [i.e., cluster 4 (*p* = 0.0000012), cluster 6 (*p* = 1.2e–156), and cluster 7 (*p* = 0)] were significant. Time course clustering algorithm: STEM, *p* < 0.05. Gene expression heatmaps of cluster 4 **(B)**, cluster 6 **(C)**, and cluster 7 **(D)**, which contained 3,339, 6,355, and 9,773 genes, respectively. Con: control population, *n* = 6; UC: ulcerative colitis patients, *n* = 12; and CD: Crohn’s disease patients, *n* = 5.

Functional genomics network analysis is a powerful tool for identifying the key regulatory networks involved in IBD progression (Peters et al., 2017). Here, gene expression clustering analysis revealed that the intestinal mucosa signaling network was comprised of eight clusters based on differential expression in control population and UC and CD patients (Figure 2A). Three clusters, i.e., cluster 4 (3,339 genes, *p* = 0.0000012), cluster 6 (6,355 genes, *p* = 1.2e–156), and cluster 7 (9,773 genes, *p* = 0), were significant according to the differential transcription levels in the control population and UC and CD patients. Cluster 6 genes were highly expressed in the intestinal mucosa of both UC and CD patients, whereas cluster 7 and 4 genes showed a gradual decrease in expression in UC patients (Figures 2B–D). Thus, these findings indicate that intestinal mucosa signaling activity was higher in CD patients than in UC patients.

### Environmental Factor Correlation Analysis Reveals Bacterial Community-Matched Autophagy Signaling in Intestinal Mucosa of IBD Patients

**Figure 3:**
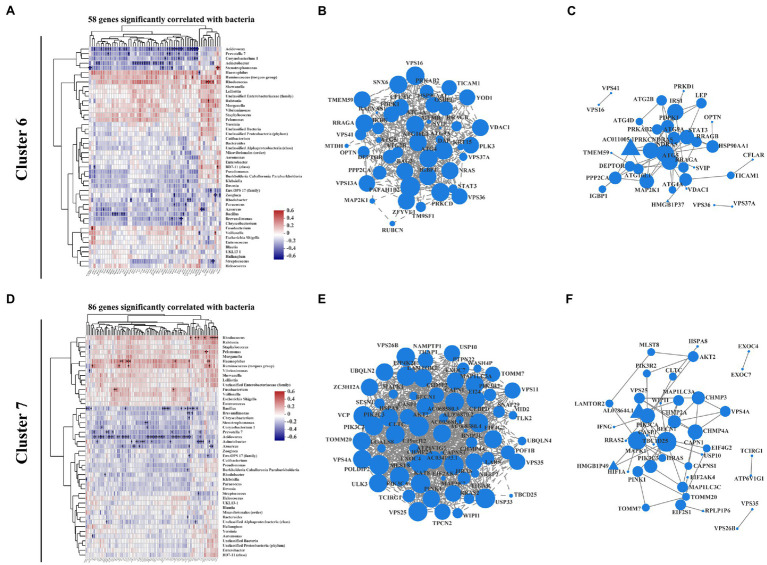
Correlation pattern between intestinal mucosa-colonizing microbiota and host autophagy signaling in IBD patients. Spearman correlation heatmap shows that 58 autophagy-related genes in cluster 6 **(A)** and 86 autophagy-related genes in cluster 7 **(D)** were significantly correlated with intestinal mucosa-colonizing bacteria (top 47). All bacteria were named to genus level unless otherwise noted in brackets. Expression correlation network of bacteria-patterned autophagy genes in cluster 6 **(B)** and cluster 7 **(E)**. Circle area is positively correlated with number of connected genes. Correlation network was constructed based on Spearman rank correlation coefficients (∣Spearman Coef∣ ≥ 0.8, *p* < 0.05). Multiple testing correction: Benjamini and Hochberg (BH). Protein–protein interaction network of bacteria-patterned autophagy genes in cluster 6 **(C)** and cluster 7 **(F)**. Circle or equilateral triangle areas are positively correlated with number of connected genes. Interaction between circle-labeled genes with others has been reported. Interaction between equilateral triangle-labeled genes with others was predicted based on primary structure of gene-coding proteins. *p* ≤ 0.05. Control population: *n* = 6; UC patient: *n* = 12; and CD patient: *n* = 5.

Increasing studies emphasize that crosstalk between the mucosal microbiome and host signaling network greatly affects IBD progression and clinical outcome (Morgan et al., 2015; Ryan et al., 2020). Recent review work provides new insights of the interplay between autophagy and intestinal bacteria and suggested that IBD-associated autophagy alleles and their interactions with environmental triggers, such as resident microbiota, are crucial for developing new therapeutic strategies for IBD treatment (Larabi et al., 2020). Thus, we performed correlation analysis between intestinal mucosa-colonizing bacteria and autophagy gene expression levels in clusters 6 and 7, which covered most KEGG autophagy-related genes (Supplementary Figure S7). In this analysis, 144 autophagy genes (58 in cluster 6 and 86 in cluster 7) were significantly correlated with intestinal mucosa-colonizing bacteria (Figures 3A,D). Gene expression heatmap analysis showed that these bacteria-correlated autophagy genes were more highly expressed in CD patients than in UC patients (Supplementary Figure S8). Furthermore, these autophagy genes not only formed a correlation network, but also a PPI network. For example, bacteria-correlated autophagy genes Autophagy related 4A cysteine peptidase (*ATG4A*), Autophagy related 5 (*ATG5*), *ATG16L1*, Autophagy related 9A (*ATG9A*), Signal transducer and activator of transcription 3 (*STAT3*), NRAS proto-oncogene, GTPase (*NRAS*), and Heat shock protein 90 alpha family class A member 1 (*HSP90AA1*) in cluster 6 (Figures 3B,C) and Beclin 1 (*BECN1*), Mitogen-activated protein kinase 1 (*MAPK1*), Phosphatidylinositol 3-kinase catalytic subunit type 3 (*PIK3C3*), Phosphatidylinositol-4,5-bisphosphate 3-kinase catalytic subunit alpha (*PIK3CA*), Charged multivesicular body protein 2A (*CHMP2A*), Charged multivesicular body protein 3 (*CHMP3*), Charged multivesicular body protein 4A (*CHMP4A*), Vacuolar protein sorting 4 homolog A (*VPS4A*), Vacuolar protein sorting 25 homolog (*VPS25*), Translocase Of outer mitochondrial membrane 20 (*TOMM20*), and AKT serine/threonine kinase 2 (*AKT2*) in cluster 7 (Figures 3E,F) were core members of both the correlation and interaction networks.

The above bacteria could also be clustered into two groups, i.e., those showing positive correlations with autophagy genes in both cluster 6 and cluster 7 (e.g., *Haemophilus*, *Ruminococcus torques*, and *Rhodococcus*) and those showing negative correlations (e.g., *Bacillus*, *Acidovorax*, *Acinetobacter*, and *Stenotrophomonas*; Figures 3A,D). It is worth noting that most of the intestinal mucosa-colonizing bacteria showing positive correlation with the above autophagy genes have been reported in previous IBD studies (Goyal et al., 2018; Soltys et al., 2020). For those bacteria negatively correlated with the autophagy genes, *Bacillus* is recognized as a probiotic bacterium in IBD prevention and therapy (Ghavami et al., 2020; Shinde et al., 2020; Zhang et al., 2020; Liu et al., 2021), but the remaining bacteria (e.g., *Acidovorax*, *Acinetobacter*, and *Stenotrophomonas*) were first reported to involve in IBD progression. Interestingly, the co-occurrence network indicated that low-abundance intestinal mucosa-colonizing bacteria were negatively and densely associated with autophagy genes, whereas high-abundance bacteria were positively and sparsely associated with autophagy genes (Supplementary Figure S6).

The autophagy genes in cluster 4, which were highly expressed in CD patients but not in UC patients compared with control population, showed more positive correlations and fewer negative correlations with the intestinal mucosa-colonizing bacteria than found in clusters 6 and 7. The positive and negative correlations were represented by *Haemophilus*, *Ruminococcus torques*, and *Rhodococcus*, and *Bacillus*, *Acidovorax*, *Acinetobacter*, and *Stenotrophomonas*, respectively. These cluster 4 bacteria-correlated autophagy genes also showed correlation and interaction networks, both of which dominated by Tumor protein P53 (*TP53*), Stimulator of interferon response CGAMP interactor 1 (*STING*), and interferon gamma inducible protein 16 (*IFI16*). These results are highly consistent with previous study suggesting that STING senses bacterial viability to orchestrate autophagy (Moretti et al., 2017). The cluster 4 co-occurrence network also showed more positive correlations between autophagy genes and intestinal mucosa-colonizing bacteria (e.g., *Haemophilus*, *Ruminococcus torques*, *Enterococcus*, *Veillonella*, *Escherichia*, and *Shigella*), but strong negative correlations between *Bacillus* and *Streptococcus* and autophagy genes [e.g., Ring finger protein 41 (*RNF41*), *BNIP3P5*, and Interleukin 10 (*IL10*); Supplementary Figure S5]. However, this correlation only existed in CD patients. Although similar correlations between mucosa-colonizing bacteria and autophagy activity have been reported (Nakagawa et al., 2004; Sudhakar et al., 2019; Wu et al., 2019), we used clinical biopsies to explore real crosstalk between mucosal bacteria and autophagy genes during IBD progression and to clarify the complex signaling pathways connecting intestinal microbes and host disease pathogenesis.

### Autophagy-Induced ER Stress Is Correlated With Intestinal Mucosa-Colonizing Bacteria in IBD Patients

**Figure 4:**
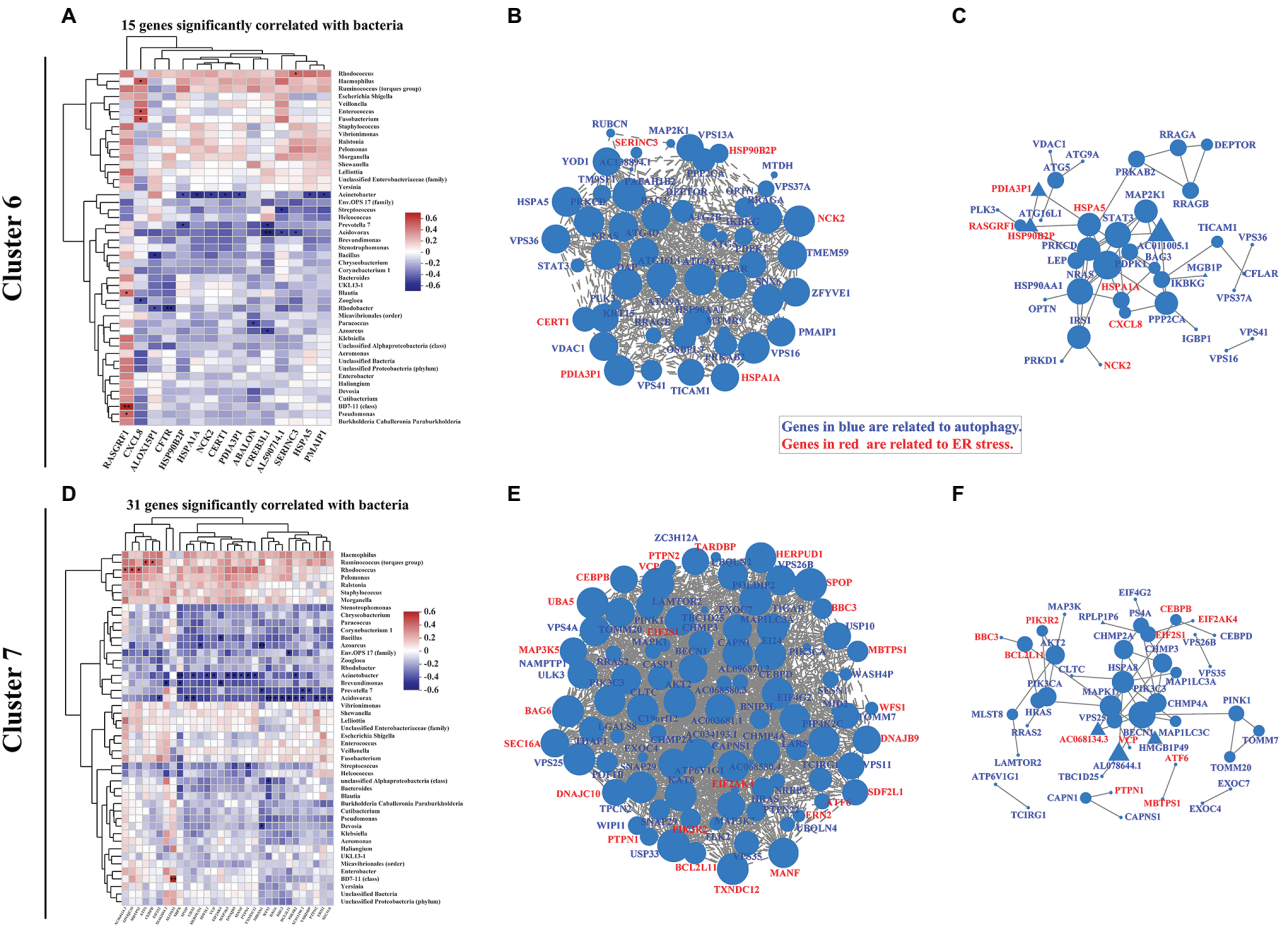
Correlation pattern of endoplasmic reticulum (ER) stress with intestinal mucosa-colonizing bacterial community and autophagy signaling in IBD patients. Spearman correlation heatmap shows 15 ER stress-related genes in cluster 6 **(A)** and 31 ER stress-related genes in cluster 7 **(D)** were significantly correlated with intestinal mucosa-colonizing bacteria (top 47). All bacteria were named to genus level unless otherwise noted in brackets. * 0.01 < *p* ≤ 0.05, ** 0.001 < *p* ≤ 0.01, and *** *p* ≤ 0.001. Correlation network of both autophagy signaling and ER stress-related genes in cluster 6 **(B)** and cluster 7 **(E)**. Autophagy signaling genes and ER stress genes are labeled in blue and red, respectively. Circle area is positively correlated with number of connected genes. Correlation network was constructed based on Spearman rank correlation coefficients (∣Spearman Coef∣ ≥ 0.8, *p* < 0.05). Multiple testing correction: BH. Interaction network of autophagy signaling and ER stress genes in cluster 6 **(C)** and cluster 7 **(F)**. Circle or equilateral triangle areas are positively correlated with number of connected genes. Interaction between circle-labeled genes with others has been reported. Interaction between equilateral triangle-labeled genes with others was predicted based on primary structure of gene-coding proteins. Control population: *n* = 6; UC patient: *n* = 12; and CD patient: *n* = 5.

Moretti et al. (2017) found that STING initiates autophagy after bacterial infection and that this autophagy is mediated by ER stress. ER stress also regulates autophagy processes during IBD progression (Kaser and Blumberg, 2009; Kaser and Blumberg, 2011; Grootjans et al., 2019). According to our analysis, 15 ER stress-related genes in cluster 6 were significantly correlated with intestinal mucosa-colonizing bacteria (Figure 4A; Supplementary Figure S12A), and the main correlations were presented with a co-occurrence network [e.g., negative correlation between *Acinetobacter* and Heat shock protein family A (Hsp70) member 1A (*HSPA1A*); Supplementary Figure S10A]. ER stress-related genes in cluster 6, such as *HSPA1A*, Heat shock protein family A (Hsp70) member 5 (*HSPA5*), Heat shock protein 90 beta family member 2 (*HSP90B2I*), Protein disulfide isomerase family A member 3 pseudogene 1 (*PDIA3P1*), and Phorbol-12-myristate-13-acetate-induced protein 1 (*PMAIP1*), formed correlation and interaction networks (Supplementary Figures S10B,C). In addition, 31 ER stress-related genes in cluster 7 were significantly correlated with mucosal bacteria (Figure 4D; Supplementary Figure S12B), and formed correlation and interaction networks (Supplementary Figures S10E,F). The main correlations were also presented with a co-occurrence network (e.g., negative correlation of *Acidovorax* with BCL2 like 11 (*BCL2L11*) and Wolframin ER transmembrane glycoprotein (*WFS1*) in cluster 7; Supplementary Figure S10D). The correlation and interaction networks of ER stress-related genes in cluster 7 mainly consisted of *Activating transcription factor 6* (*ATF6*), Homocysteine inducible ER protein with ubiquitin like domain 1 (*HERPUD1*), BAG cochaperone 6 (*BAG6*), BCL2 binding component 3 (*BBC3*), Mesencephalic astrocyte derived neurotrophic factor (*MANF*), DnaJ heat shock protein family (Hsp40) member B9 (*DNAJB9*), *BCL2L11*, Membrane bound transcription factor peptidase, site 1 (*MBTPS1*), Eukaryotic translation initiation factor 2 subunit alpha (*EIF2S1*), Valosin containing protein (*VCP*), Mitogen-activated protein kinase kinase kinase 5 (*MAP3K5*), and Thioredoxin domain containing 12 (*TXNDC12*; Supplementary Figures S10E,F). HSPA5-induced ATF6 activity is a typical representative of ER stress signaling in the intestinal epithelium (Stengel et al., 2020) that bridging clusters 6 and 7 profile in this study. Furthermore, combinative analysis of both correlation and interaction network, also exhibited the complexity of ER stress-autophagy signaling cascade in IBD (Figures 4B,C,E,F). In addition, we also found similar ER stress signaling based on KEGG functional pathway analysis (Supplementary Figure S11).

In cluster 4, six ER stress-related genes [i.e., endoplasmic reticulum protein 27 (*ERP27*), NHL repeat containing E3 ubiquitin protein ligase 1 (*NHLRC1*), Fc gamma receptor IIb (*FCGR2B*), Stanniocalcin 2 (*STC2*), Caspase 17, pseudogene (*CASP17P*), and ChaC glutathione specific gamma-glutamylcyclotransferase 1 (*CHAC1*)] were significantly correlated with intestinal mucosa-colonizing bacteria and were highly expressed in CD patients but not in UC patients compared with the control population. The co-occurrence network showed that both *STC2* and *FCGR2B* were negatively and positively correlated with Proteobacteria (*Acinetobacter* and *Klebsiella*), respectively. Interestingly, both *STC2* and *FCGR2B* are involved in ER stress activities during IBD progression (Franke et al., 2016; Coope et al., 2019). In addition, co-occurrence network analyses also linked autophagy signaling of *STING* and *IL10* with the ER stress activities of *CASP17P* and *FCGR2B* (Supplementary Figure S9).

### ER Stress-Related Bile Acid Production Signaling Is Correlated With Mucosa-Colonizing Bacteria in IBD Patients

**Figure 5:**
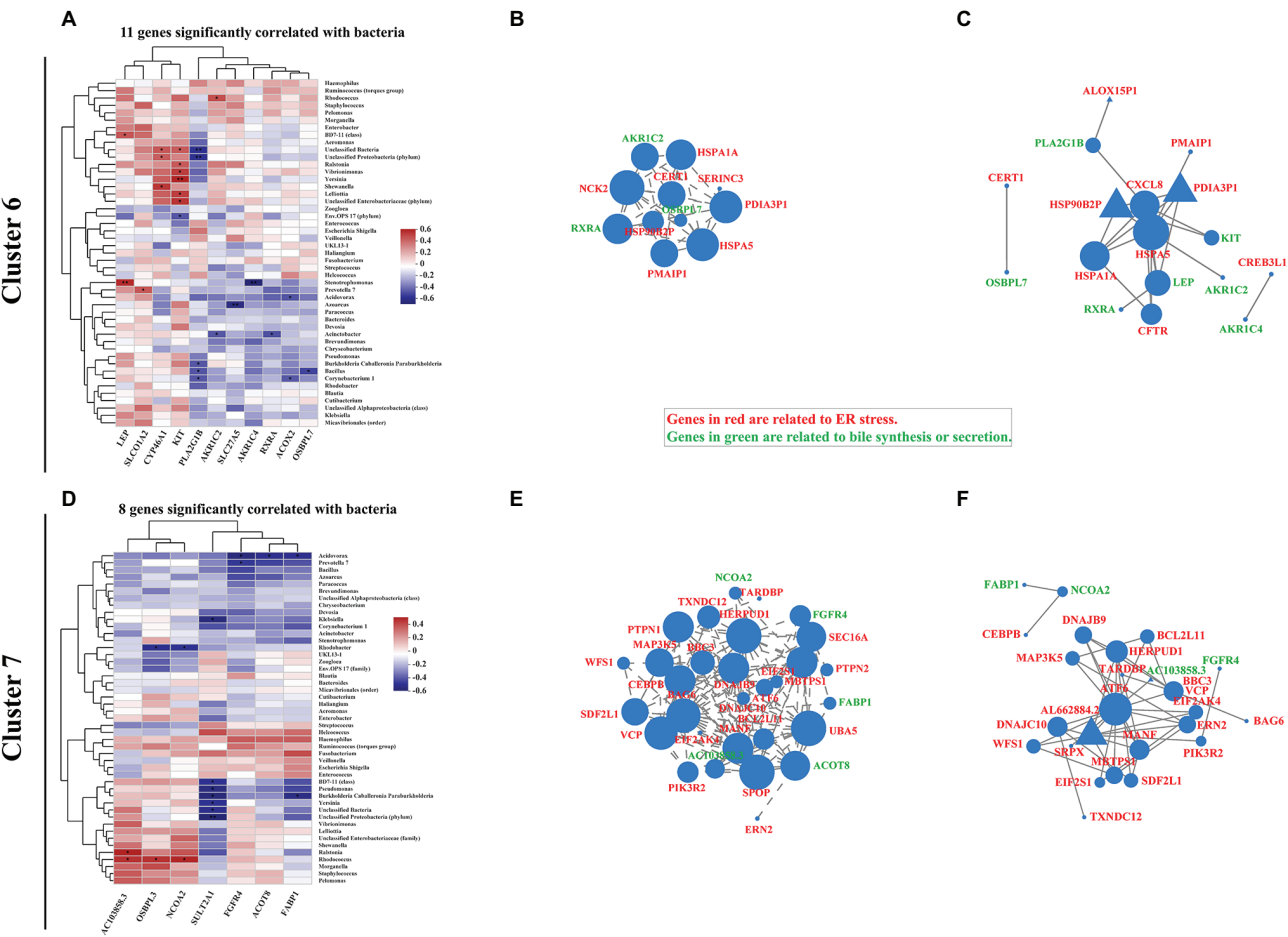
Correlation pattern of bile acid production with intestinal mucosa-colonizing bacteria community and ER stress in IBD patients. Spearman correlation heatmap shows 11 bile-related genes in cluster 6 **(A)** and eight bile-related genes in cluster 7 **(D)** were significantly correlated with intestinal mucosa-colonizing bacteria (top 47). All bacteria were named to genus level unless otherwise noted in brackets. * 0.01 < *p* ≤ 0.05, ** 0.001 < *p* ≤ 0.01, and *** *p* ≤ 0.001. Correlation network of both ER stress- and bile acid production-related genes in cluster 6 **(B)** and cluster 7 **(E)**. ER stress- and bile acid production-related genes are labeled in red and green, respectively. Circle area is positively correlated with number of connected genes. Correlation network was constructed based on Spearman rank correlation coefficients (∣Spearman Coef∣ ≥ 0.8, *p* < 0.05). Multiple testing correction: BH. Interaction network of ER stress- and bile acid production-related genes in cluster 6 **(C)** and cluster 7 **(F)**. Circle or equilateral triangle areas are positively correlated with number of connected genes. Interaction between circle-labeled genes with others has been reported. Interaction between equilateral triangle-labeled genes with others was predicted based on primary structure of gene-coding proteins. Control population: *n* = 6; UC patient: *n* = 12; and CD patient: *n* = 5.

Increasing evidence indicates that intestinal dysbiosis induces bile acid dysmetabolism and consequent IBD progression (Duboc et al., 2013; Lloyd-Price et al., 2019; Quinn et al., 2020; Sinha et al., 2020). Bacterial-driven bile acid metabolites have also been investigated to describe the pathophysiological basis of bacteria-bile acid associations (Long et al., 2017; Lavelle and Sokol, 2020). Here, we expanded the association between bile acid production-related genes and intestinal mucosa-colonizing bacteria. Eleven genes [e.g., Leptin (*LEP*), cytochrome P450 family 46 subfamily A member 1 (*CYP46A1*), KIT proto-oncogene, receptor tyrosine kinase (*KIT*), Phospholipase A2 group IB (*PLA2G1B*), and Retinoid X receptor alpha (*RXRA*)] in cluster 6 and eight genes [e.g., Sulfotransferase family 2A member 1 (*SULT2A1*), Fibroblast growth factor receptor 4 (*FGFR4*), and fatty acid binding protein 1 (*FABP1*)] in cluster 7 were significantly correlated with the intestinal mucosa-colonizing bacteria (Figures 5A,D). Similar to autophagy and ER stress signaling, genes in both cluster 6 and cluster 7 formed correlation and interaction networks, dominated by *RXRA* (Supplementary Figures S14B,C) and *FABP1* (Supplementary Figures S14D,E), respectively. Notably, *KIT* and *PLA2G1B* in cluster 6 (Supplementary Figure S14A) and *SULT2A1* in cluster 7 (Supplementary Figure S14D) showed strong correlations with the intestinal mucosa-colonizing bacteria. Bile-regulated lipid metabolism can induce ER stress in intestinal epithelial cells and intestinal bacteria modulate this metabolic process (Ko et al., 2020). Here, we identified bile acid-related genes in cluster 6 [*RXRA*, *Aldo-keto reductase family 1 member C2* (*AKR1C2*), and Oxysterol binding protein like 7 (*OSBPL7*)] and cluster 7 [*FGFR4*, Nuclear receptor coactivator 2 (*NCOA2*), and *FABP1*], which were all significantly correlated with intestinal mucosa-colonizing bacteria and formed both correlation and interaction networks with ER stress signaling (Figures 5B,C,E,F). KEGG functional analysis also revealed the upregulation of genes related to bile biosynthesis (Supplementary Figure S15) and secretion (Supplementary Figure S16). Furthermore, the expression differentiation of bile acid-producing gene between UC and CD patients increased in cluster 7 than in cluster 6 (Supplementary Figure S17).

In the other hand, the bacteria that correlated with intestinal mucosa bile acid signaling, mainly comprised of Proteobacteria. The correlations between *Stenotrophomonas*, *Acinetobacte*r, *Bacillus*, and *Corynebacterium* and cluster 6 genes (Figure 5A; Supplementary Figure S14A) and *Rhodococcus* and *Acidovorax* and cluster 7 genes (Figure 5B; Supplementary Figure S14B), exhibited the most significant correlations with host bile acid signaling. In addition, *FGF19* in cluster 4, which is reported to modulate the connection between intestinal microbiota and host inflammation (Gadaleta et al., 2020), was correlated with multiple intestinal bacteria, including *Staphylococcus*, *Pelomonas*, *Haemophilus*, *Ruminococcus torques*, and *Bacteroides* (Supplementary Figure S13).

## Discussion

Increasing studies focus on the mucosal microbiome and local host immune activities in IBD patients (Howell et al., 2018; Lloyd-Price et al., 2019; Ryan et al., 2020) and the role of autophagy in IBD progression, especially in CD patients (Khor et al., 2011; Larabi et al., 2020). To date, however, no studies have reported on how mucosal bacteria interact with host autophagy activity based on biopsy samples even that obviously hold a great interest of precise diagnosis and therapy of IBD. Here, we used intestinal mucosal biopsies to elucidate the interaction network between mucosal bacteria and host autophagy signaling, as this network can reveal the bacterial infection-related pathogenesis of IBD and highlight potential and precise therapeutic targets for IBD.

Our results showed that active mucosal signaling, including autophagy, could be divided into cluster 4, 6, and 7 based on signaling extensity in UC patients. In general, all genes in the three clusters were highly activated in CD patients, but only genes in cluster 6 were highly expressed in UC patients. In addition, genes in cluster 7 showed increased expression in UC patients compared with that in the control population. The genes in cluster 4 also showed the same activities in UC patients and control population. These results are consistent with previous study showing that autophagy genes are pivotal for intestinal homeostasis and antimicrobial function in IBD, especially CD (Larabi et al., 2020).

In our study, UC patients exhibited greater disturbance in mucosal microbial diversity, community phenotype, and underlying functional spectrum, whereas CD patients exhibited greater autophagy and associated signaling cascades. These differences may have originated from the different pathological durations of UC and CD in patients. The long disease course in CD patients can strongly induce autophagy signaling to fight pathogenic microbial invasion. In another hand, pathogenic microbial invasion can induce CD progression, whereas diffusion behavior tends to increase UC progression (Mirsepasi-Lauridsen et al., 2019). Certainly, mucosal biopsies are ideal samples to determine bacterial diffusion behavior. Previous studies using fecal samples found that CD patients show more extensive microbial community disturbance than UC patients (Halfvarson et al., 2017; Pascal et al., 2017). Based on our findings, we suggest that CD patients continuously repair their gut defenses and establish new gut immune hemostasis during the long-term pathological progression of the disease, which is different from normal states and UC patients.

We also found that most dominant bacteria were positively correlated with autophagy genes and related signaling cascades, whereas less abundant bacteria showed negative correlations. Dominant bacteria may form stronger community structures that can withstand serious environmental shifts or may change into opportunistic pathogens for survival. In contrast, low-abundance bacteria communities may be more sensitive to environmental shift, and therefore tightly related to host metabolism and healthy conditions and finally process a high sensitivity and great value for intestinal disease diagnosis and therapy, including IBD. However, the low-abundance bacteria also brought some uncertainty. For example, *Acidovorax* (<0.2% of total abundance) was negatively correlated with most autophagy genes and related signaling cascades, even these genes or signaling play an adversarial role in autophagy. In addition, we observed several bacterial species that were not significantly correlated with any host genes (data not shown); it was difficult to explain how they survive under severe immune stress from host and fierce competition from other microbes if they do not clearly show cooperative (or confrontational) properties.

Our study has several limitations. Both the recruited patients (average age > 45) and control population (average age > 35) were older than participants in other studies, which is likely due to the consciousness deficiency of IBD risk in the local young population. In this study, the microbiota-correlated autophagy genes concurrently showed a strong correlation with ER stress. Integrating previous study (Kökten et al., 2018), the autophagy in our study could be either macroautophagy or chaperone-mediated autophagy, but the specific subtype was not clear and needs to be further explored. The relationship between mucosal bacteria and host autophagy activity needs more mucosa biopsies to conform their correlation profiles, but our matched analysis of microbiome and transcriptome from same subjects and location still drew an interactive map of bacteria-autophagy-IBD progression and could provide more valuable diagnostic and therapeutic targets based on autophagy mechanism.

## Conclusion

In conclusion, we found that UC patients exhibited more severe dysbiosis and the functional phenotype of intestinal mucosa-colonizing bacteria, whereas CD patients exhibited more active autophagic signaling compared to the controls. Dominant bacteria and low-abundance bacteria showed positive and negative correlations with host autophagy genes, respectively. In addition, correlation and interaction networks were found between bile acid production and ER stress, with the latter showing an interesting interaction with autophagy activity. Thus, the present study elucidated how the intestinal mucosa-colonizing bacterial community interacts with host bile/ER stress/autophagy signaling cascades during IBD progression, which could aid in disease diagnosis and autophagy-targeted therapy.

## Data Availability Statement

The datasets presented in this study can be found in online repositories. The names of the repository/repositories and accession number(s) can be found at: https://www.ncbi.nlm.nih.gov/, PRJNA797846.

## Ethics Statement

The studies involving human participants were reviewed and approved by the Medical Ethics Board of the First People’s Hospital of Yunnan Province (GXBSC-2021001, 2021 updated). The patients/participants provided their written informed consent to participate in this study.

## Author Contributions

WW and JG designed the project and reviewed and revised the final version of the manuscript. WW and ZL analyzed the 16S rRNA sequencing data. LZ, WY, HZ, CY, TH, PW, and JG collected mucosal biopsies and clinical documents. WW, ZL, and JG finished the draft. WW, LZ, PW, and JG supervised the study and rendered foundation supports. All authors contributed to the article and approved the submitted version.

## Funding

This work was supported by the National Natural Science Foundation of China (81860437 and 82160514), the Yunnan Province Innovation Team of Intestinal Microecology-Related Disease Research and Technological Transformation (202005AE160010), Eminent Doctors Program of Yunnan Province (YNWR-MY-2019-072), Yunnan Digestive Endoscopy Clinical Medical Center Foundation [2X2019-01-02]-(2019LCZXKF-XH05, 2020LCZXKF-XH01, 2021LCZXXF-XH01, and 2021LCZXXF-XH15), and Fundamental Research Projects of Yunnan Province (202101AT070275, 202101AY070001-236, and 2018FE001-130).

## Conflict of Interest

The authors declare that the research was conducted in the absence of any commercial or financial relationships that could be construed as a potential conflict of interest.

## Publisher’s Note

All claims expressed in this article are solely those of the authors and do not necessarily represent those of their affiliated organizations, or those of the publisher, the editors and the reviewers. Any product that may be evaluated in this article, or claim that may be made by its manufacturer, is not guaranteed or endorsed by the publisher.

## Figures and Tables

**Table:** 

AKR1C2	Aldo-keto reductase family 1 member C2
AKT2	AKT serine/threonine kinase 2
ATF6	Activating transcription factor 6
ATG16L1	Autophagy related 16 like 1
ATG4A	Autophagy related 4A cysteine peptidase
ATG5	Autophagy related 5
ATG9A	Autophagy related 9A
BAG6	BAG cochaperone 6
BBC3	BCL2 binding component 3
BCL2L11	BCL2 like 11
BECN1	Beclin 1
BNIP3P	BCL2 interacting protein 3 pseudogene 1
CASP17P	Caspase 17, pseudogene
CD	Crohn’s disease
CHAC1	ChaC glutathione specific gamma-glutamylcyclotransferase 1
CHMP2A	Charged multivesicular body protein 2A
CHMP3	Charged multivesicular body protein 3
CHMP4A	Charged multivesicular body protein 4A
CYP46A1	cytochrome P450 family 46 subfamily A member 1
DNAJB9	DnaJ heat shock protein family (Hsp40) member B9
EIF2S1	Eukaryotic translation initiation factor 2 subunit alpha
ER	Endoplasmic reticulum
ERP27	Endoplasmic reticulum protein 27
FABP1	Fatty acid binding protein 1
FAPROTAX	Functional annotation of prokaryotic taxa
FCGR2B	Fc gamma receptor IIb
FGFR4	Fibroblast growth factor receptor 4
HERPUD1	Homocysteine inducible ER protein with ubiquitin like domain 1
HSP90AA1	Heat shock protein 90 alpha family class A member 1
HSP90B2I	Heat shock protein 90 beta family member 2
HSPA1A	Heat shock protein family A (Hsp70) member 1A
HSPA5	Heat shock protein family A (Hsp70) member 5
IBD	Inflammatory bowel disease
IFI16	Interferon gamma inducible protein 16
IL10	Interleukin 10
KEGG	Kyoto encyclopedia of genes and genomes
KIT	KIT proto-oncogene, receptor tyrosine kinase
LEP	Leptin
MANF	Mesencephalic astrocyte derived neurotrophic factor
MAP3K5	Mitogen-activated protein kinase kinase kinase 5
MAPK1	Mitogen-activated protein kinase 1
MBTPS1	Membrane bound transcription factor peptidase, site 1
NCOA2	Nuclear receptor coactivator 2
NHLRC1	NHL repeat containing E3 ubiquitin protein ligase 1
NRAS	NRAS proto-oncogene, GTPase
OSBPL7	Oxysterol binding protein like 7
PDIA3P1	Protein disulfide isomerase family A member 3 pseudogene 1
PIK3C3	Phosphatidylinositol 3-kinase catalytic subunit type 3
PIK3CA	Phosphatidylinositol-4,5-bisphosphate 3-kinase catalytic subunit alpha
PLA2G1B	Phospholipase A2 group IB
PMAIP1	Phorbol-12-myristate-13-acetate-induced protein 1
RNF41	Ring finger protein 41
RXRA	Retinoid X receptor alpha
STAT3	Signal transducer and activator of transcription 3
STC2	Stanniocalcin 2
STING	Stimulator of interferon response CGAMP interactor 1
SULT2A1	Sulfotransferase family 2A member 1
TMEM59	Transmembrane protein 59
TOMM20	Translocase Of outer mitochondrial membrane 20
TP53	Tumor protein P53
TXNDC12	Thioredoxin domain containing 12
UC	Ulcerative colitis
VCP	Valosin containing protein
VPS25	Vacuolar protein sorting 25 homolog
VPS4A	Vacuolar protein sorting 4 homolog A
WFS1	Wolframin ER transmembrane glycoprotein

## Footnotes

^1^ https://github.com/jstjohn/SeqPrep

^2^ https://github.com/najoshi/sickle

^3^ http://ccb.jhu.edu/software/hisat2/index.shtml

^4^ https://ccb.jhu.edu/software/stringtie/index.shtml?t=example

^5^ http://deweylab.biostat.wisc.edu/rsem/

^6^ https://github.com/tanghaibao/Goatools

^7^ http://string-db.org/
