# Supplementary material for: Mucosa-Colonizing Microbiota Correlate With Host Autophagy Signaling in Patients With Inflammatory Bowel Disease
Source: Front Microbiol. 2022 May 26;13:875238. doi: 10.3389/fmicb.2022.875238 (PMC9178242; doi:10.3389/fmicb.2022.875238)
Supplement: Supplementary file 1 [file Data_Sheet_1.DOCX]

**
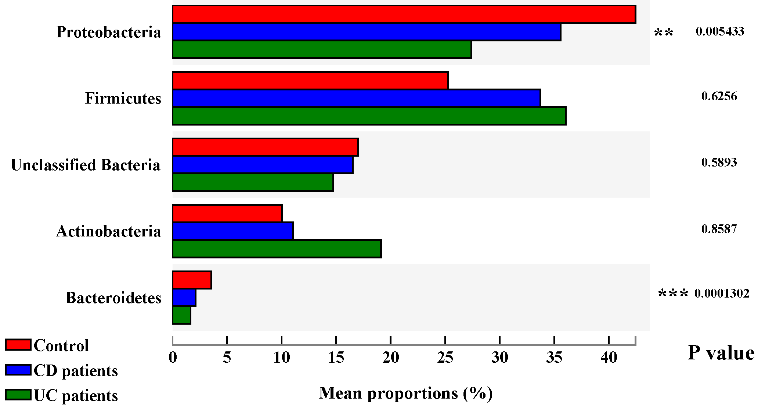
**

**Figure S1.** K**ruskal-Wallis H test compared average composition of intestinal mucosa-colonizing bacteria at phylum level**. Multiple testing correction: false discovery rate (FDR); *Post-hoc* test: Tukey-Kramer (CI = 0.95).

**
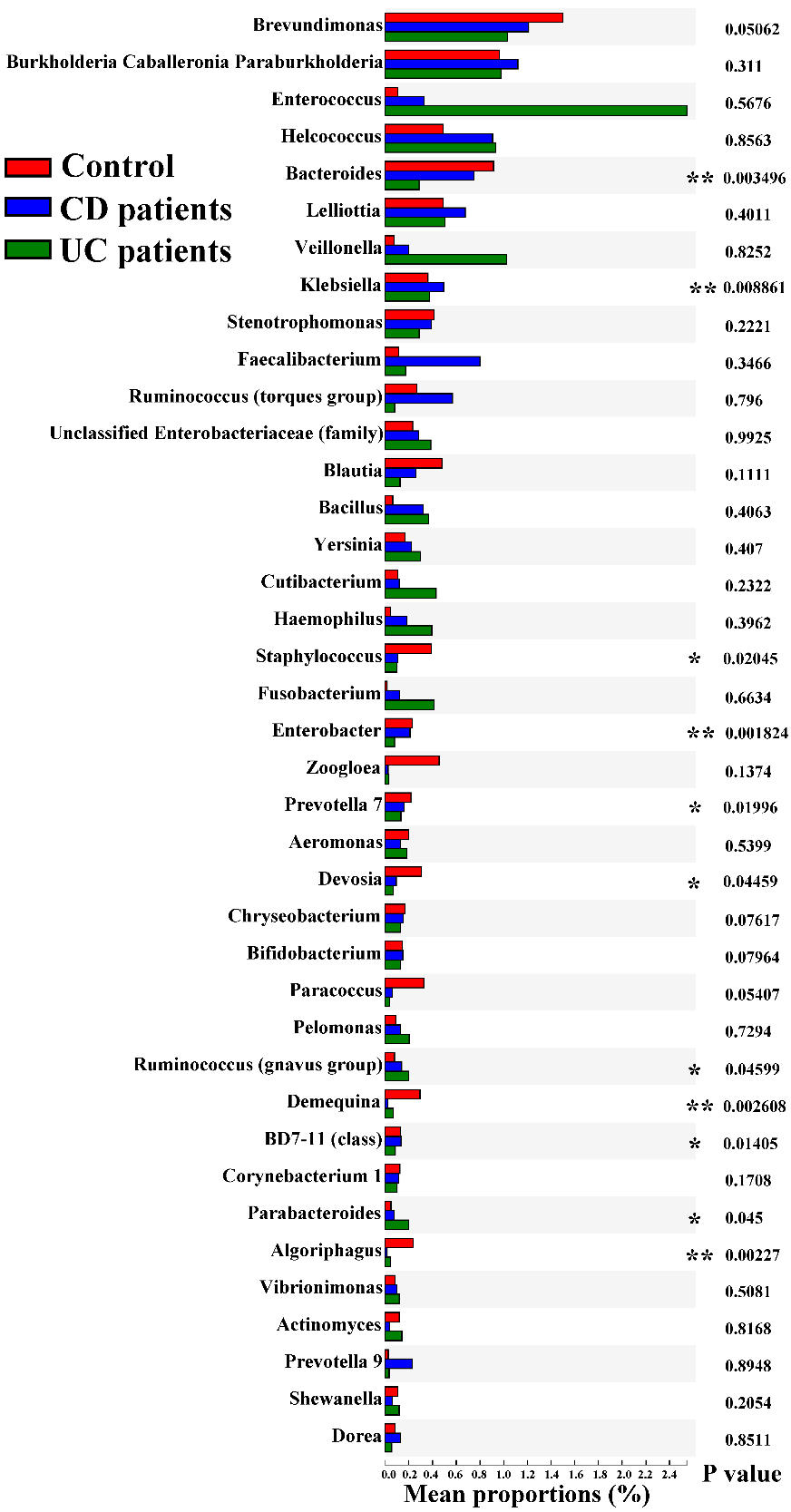
**

**Figure S2. Intestinal mucosa-colonizing bacteria (top 9 to top 47) in control population and IBD patients.** Kruskal-Wallis H test compared average bacterial composition. Multiple testing correction: FDR; *Post-hoc* test: Tukey-Kramer (CI = 0.95). * 0.01 < *P* ≤ 0.05, ** 0.001 < *P* ≤ 0.01. Control population: n = 23; CD patient: n = 26; UC patient: n = 51. All bacteria were named to genus level unless otherwise noted in brackets.


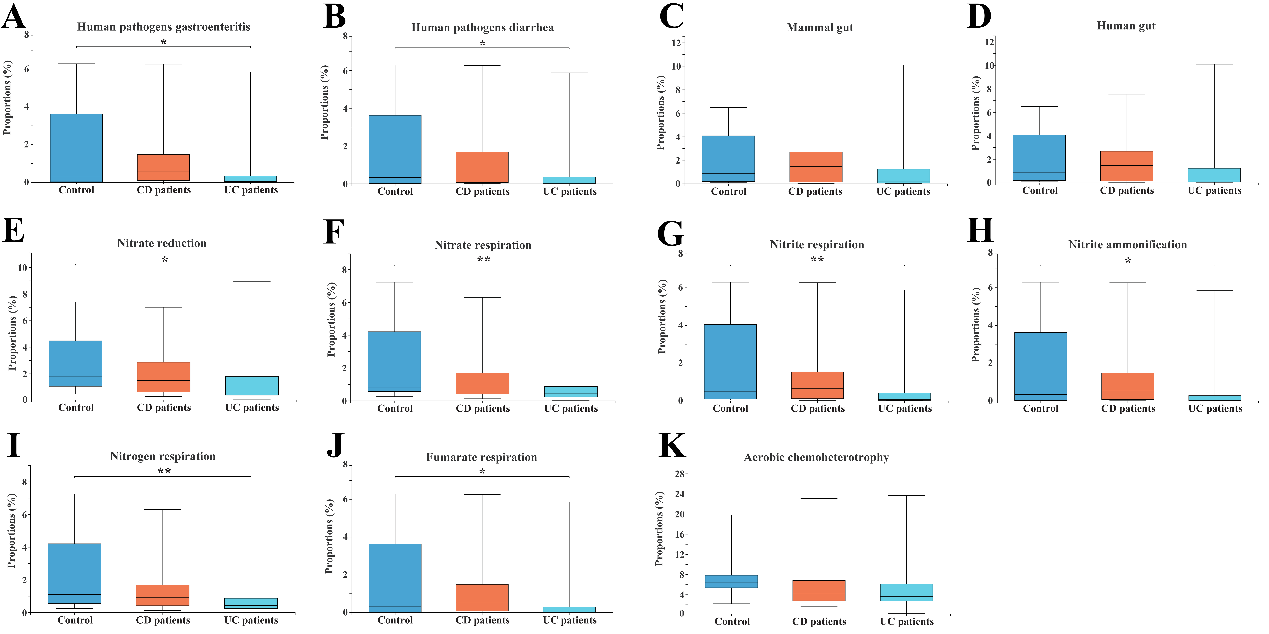


**Figure S3. Predicted bacterial functional variation from control population to IBD patients.** FAPROTAX approach predicted potential bacterial functions, including human pathogens gastroenteritis (**A**), human pathogens diarrhea (**B**), mammal gut (**C**), human gut (**D**), nitrate reduction (**E**), nitrate respiration (**F**), nitrite respiration (**G**), nitrite ammonification (**H**), nitrogen respiration (**I**), fumarate respiration (**J**) and aerobic chemoheterotrophy (**K**), used Kruskal-Wallis H test. Multiple testing correction: FDR; *Post-hoc* test: Tukey-Kramer (CI = 0.95). * 0.01 < *P* ≤ 0.05, ** 0.001 < *P* ≤ 0.01. Control population: n = 23; CD patient: n = 26; UC patient: n = 51.


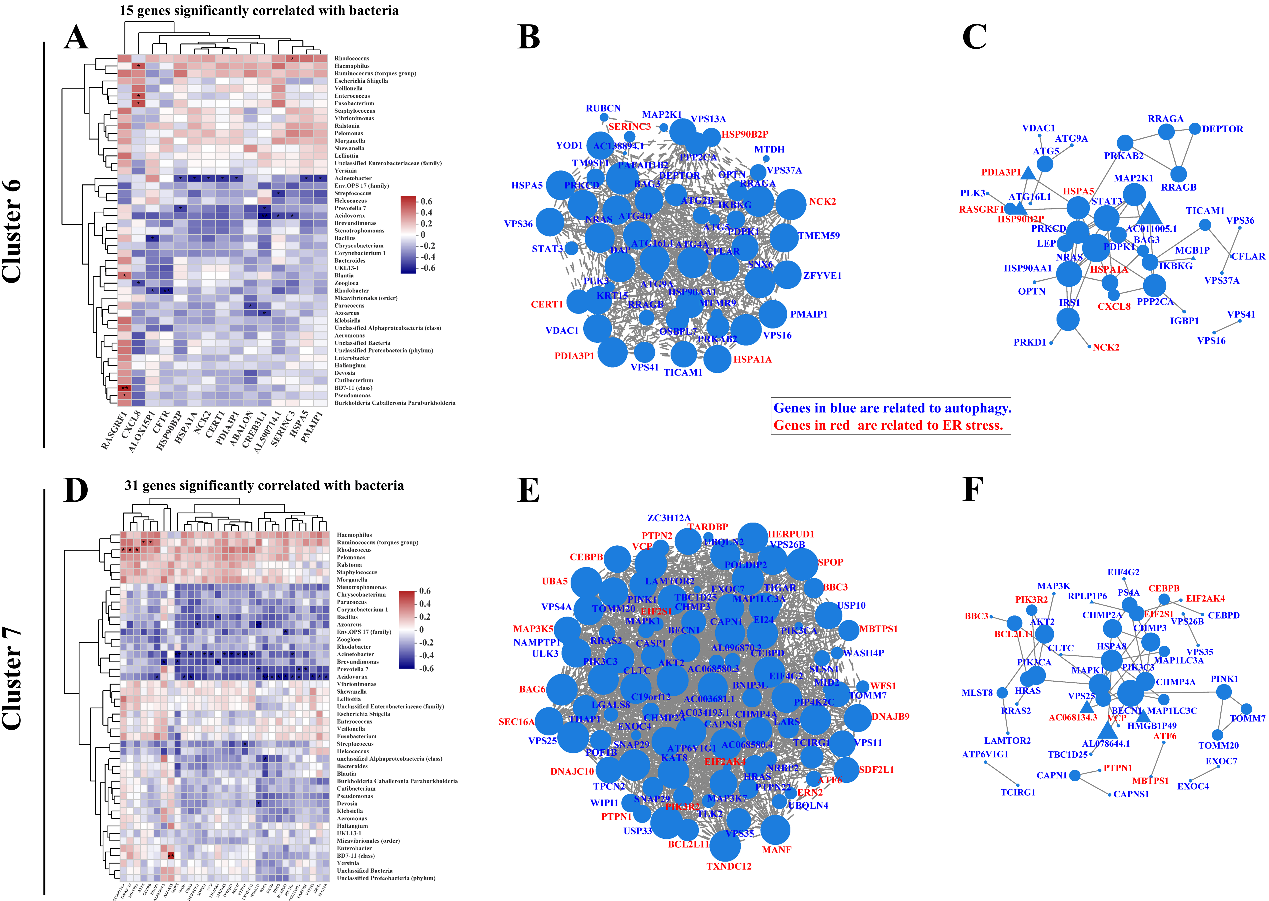


**Figure S4. Predicted bacterial community phenotypes of IBD patients.** BugBase-predicted phenotypes of oxidative stress tolerance decreased (**A**), and gram-positive phenotypes increased (**C**) in intestinal mucosa of IBD patients based on Kruskal-Wallis H test. Multiple testing correction: FDR; *Post-hoc* test: Tukey-Kramer (CI = 0.95). * 0.01 < *P* ≤ 0.05, ** 0.001 < *P* ≤ 0.01. Bacterial contributions (top 47) to increased oxidative stress tolerance (**B**) and increased gram-positive phenotypes (**D**) at genus level. Block area was positively correlated with bacterial abundance. Control population: n = 23; CD patient: n = 26; UC patient: n = 51. All bacteria were named to genus level unless otherwise noted in brackets.


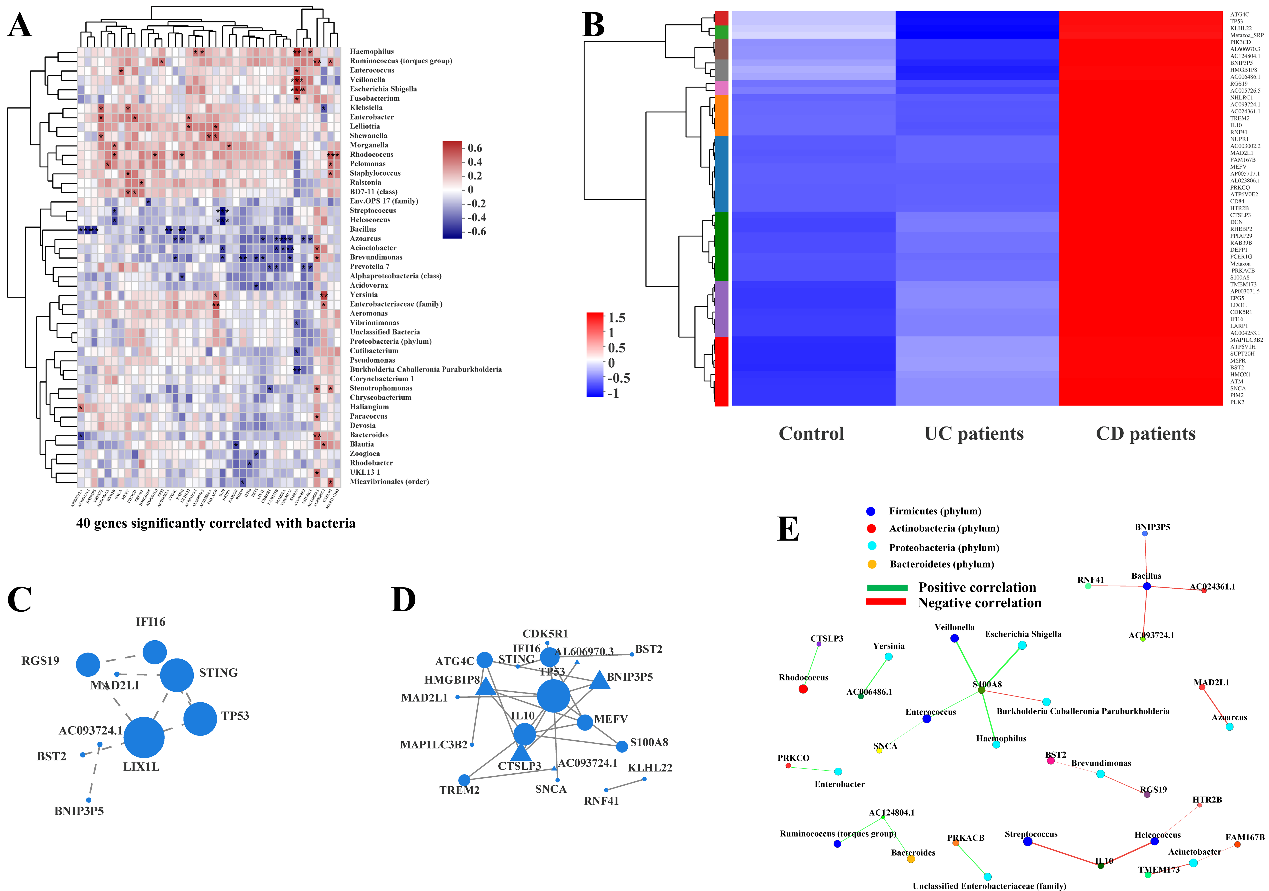


**Figure S5.** **Intestinal mucosa autophagy signaling in cluster 4 and correlation with mucosa-colonizing microbiota in IBD patients.** (**A**) Spearman correlation heatmap shows 40 autophagy-related genes in cluster 4 were significantly correlated with intestinal mucosa-colonizing bacteria (top 47). All bacteria were named to genus level unless otherwise noted in brackets. * 0.01 < *P* ≤ 0.05, ** 0.001 < *P* ≤ 0.01, *** *P* ≤ 0.001. (**B**) Gene expression heatmap of autophagy genes in cluster 4. (**C**) Correlation network of 40 autophagy-related genes in cluster 4. Correlation network was constructed based on Spearman rank correlation coefficients (∣Spearman Coef∣ ≥ 0.8, *P* < 0.05). Circle area is positively correlated with number of connected genes. Multiple testing correction: BH. (**D**) Protein-protein interaction network of bacteria-patterned autophagy genes in cluster 4. Interaction between circle-labeled genes with others has been reported. Interaction between equilateral triangle-labeled genes with others was predicted based on primary structure of gene-coding proteins. Circle or equilateral triangle areas are positively correlated with number of connected genes. (**E**) Co-occurrence network between autophagy genes in cluster 4 and patterned bacteria (top 47). Co-occurrence network was constructed based on Spearman rank correlation coefficients (∣Spearman Coef∣ ≥ 0.5, *P* < 0.05). Lines connecting different nodes indicate positive (green) or negative (red) correlation between bacteria and genes, and line diameter is positively related to correlation value. Control population: n = 6; UC patient: n = 12; CD patient: n = 5. All bacteria were named to genus level unless otherwise noted in brackets.


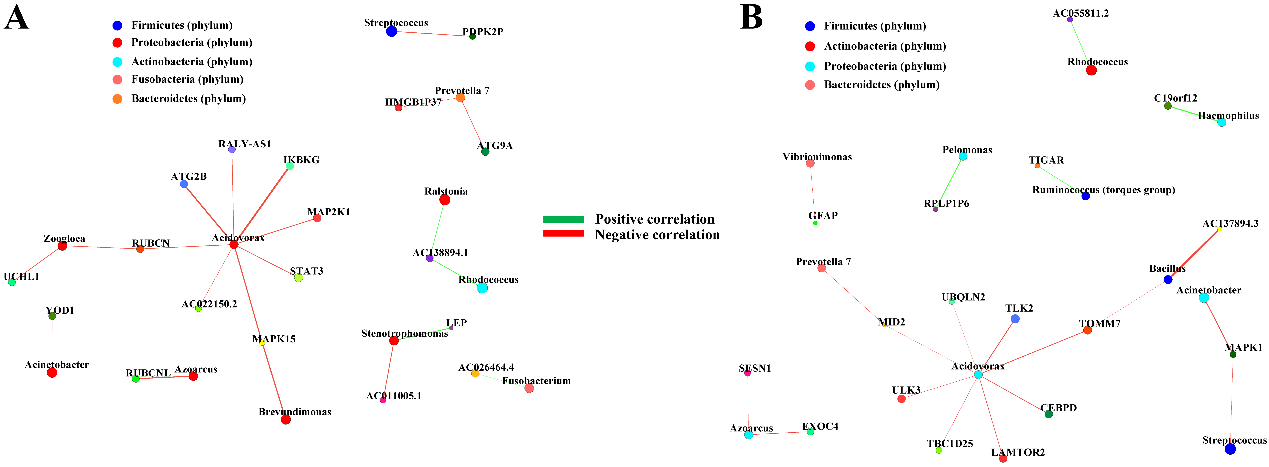


**Figure S6.** **Co-occurrence network between autophagy genes in cluster 6 (A) and cluster 7 (B) and intestinal mucosa-colonizing bacteria (top 47).** Co-occurrence network was constructed based on Spearman rank correlation coefficients (∣Spearman Coef∣ ≥ 0.5, *P* < 0.05). Lines connecting different nodes indicate positive (green) or negative (red) correlation between bacteria and genes, and line diameter is positively related to correlation value. Control population: n = 6; UC patient: n = 12; CD patient: n = 5. All bacteria were named to genus level unless otherwise noted in brackets.


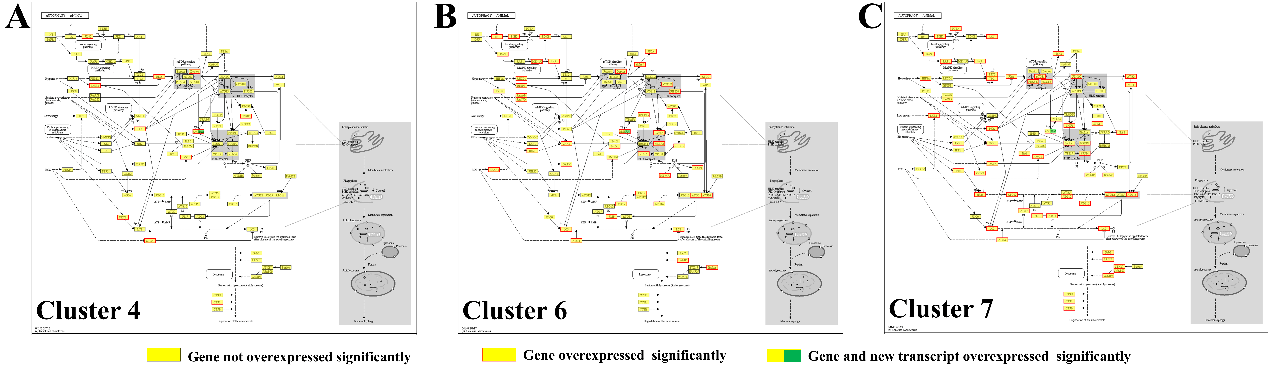


**Figure S7. KEGG autophagy enrichment analysis of cluster 4 (A), cluster 6 (B), and cluster 7 (C).** Multiple testing correction: BH. Control population: n = 6; UC patient: n = 12; CD patient: n = 5.


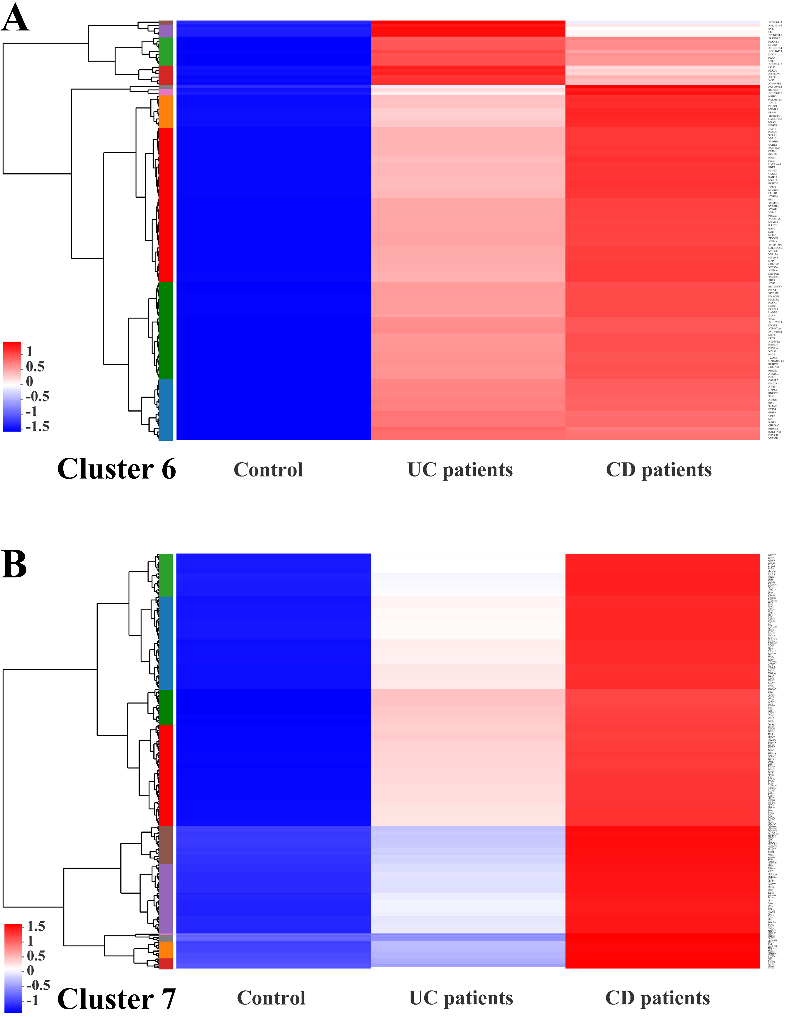


**Figure S8. Gene expression heatmaps of autophagy genes in cluster 6 (A) and cluster 7 (B) in intestinal mucosa of IBD patients.** Control population: n = 6; UC patient: n = 12; CD patient: n = 5.


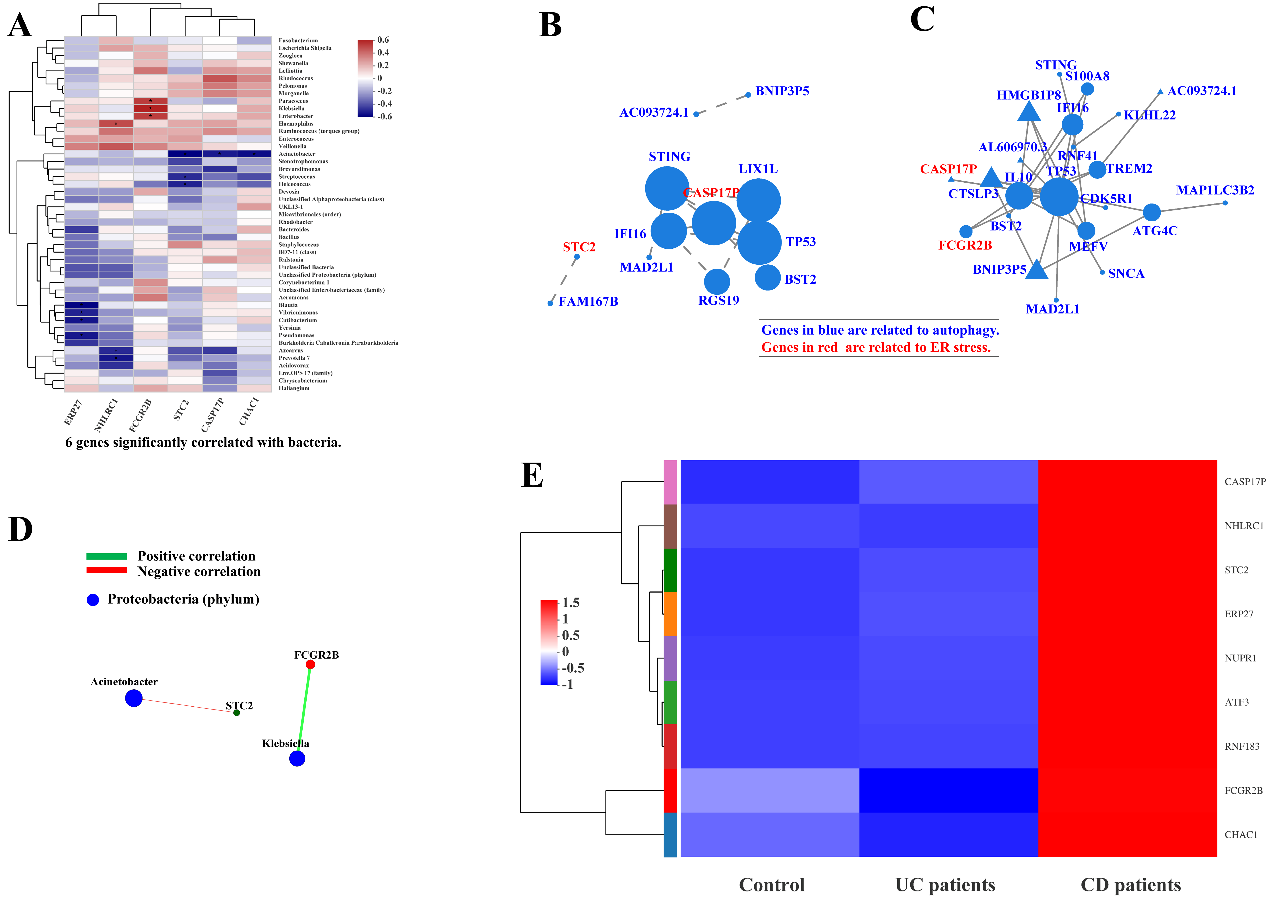


**Figure S9. ER stress activation in cluster 4 and correlation with mucosa-colonizing microbiota in IBD patients.** (**A**) Spearman correlation heatmap shows six ER stress-related genes in cluster 4 were significantly correlated with mucosa-colonizing bacteria (top 47). All bacteria were named to genus level unless otherwise noted in brackets. * 0.01 < *P* ≤ 0.05, ** 0.001 < *P* ≤ 0.01, *** *P* ≤ 0.001. (**B**) Correlation network of ER stress-related genes in cluster 4. Correlation network was constructed based on Spearman rank correlation coefficients (∣Spearman Coef∣ ≥ 0.8, *P* < 0.05). Circle area is positively correlated with number of connected genes. Multiple testing correction: BH. (**C**) Interaction network of ER stress-related genes in cluster 4. Interaction between circle-labeled genes with others has been reported. Interaction between equilateral triangle-labeled genes with others was predicted based on primary structure of gene-coding proteins. Circle or equilateral triangle areas are positively correlated with number of connected genes. (**D**) Co-occurrence network between ER stress-related genes in cluster 4 and intestinal mucosa-colonizing bacteria (top 47). Co-occurrence network was constructed based on Spearman rank correlation coefficients (∣Spearman Coef∣ ≥ 0.5, *P* < 0.05). Lines connecting different nodes indicate positive (green) or negative (red) correlation between bacteria and genes, and line diameter is positively related to correlation value. All bacteria were named to genus level unless otherwise noted in brackets. (**E**) Gene expression heatmap of autophagy genes in cluster 4. Control population: n = 6; UC patient: n = 12; CD patient: n = 5.


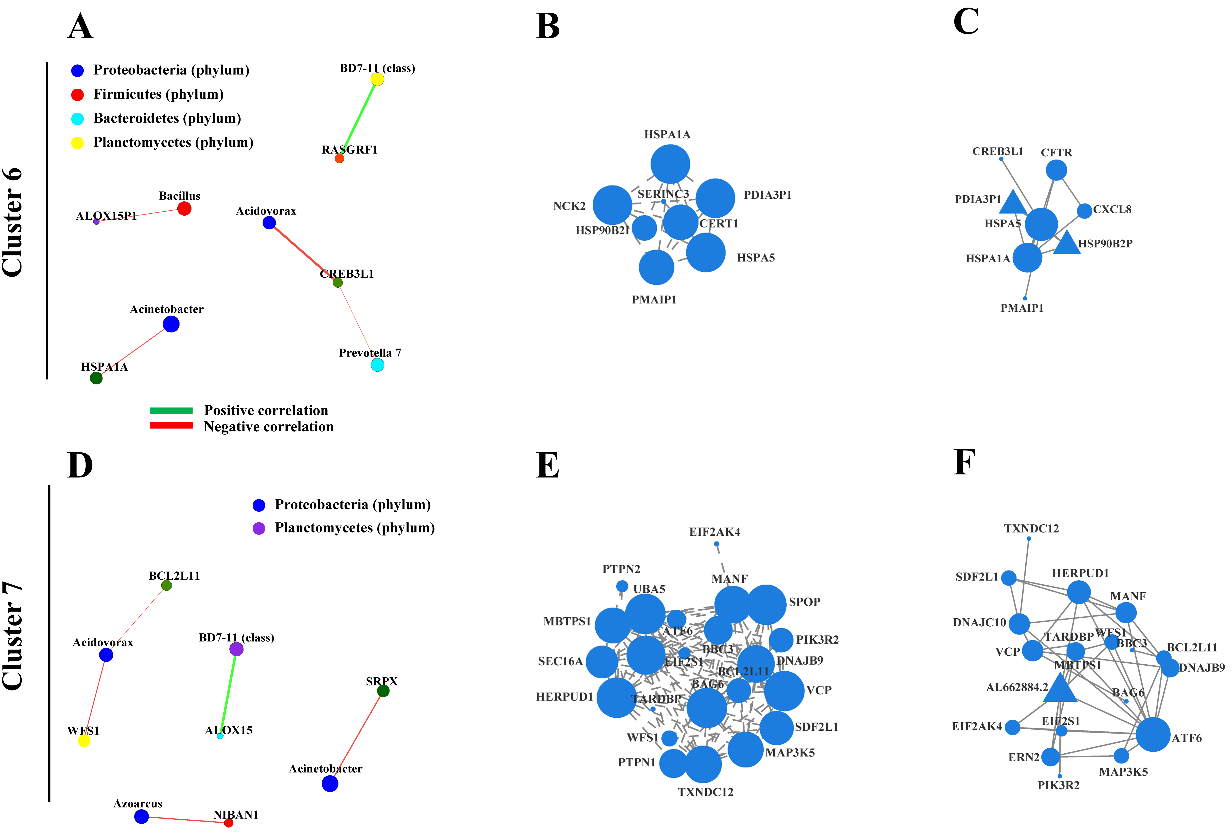


**Figure S10.** **ER stress network in intestinal mucosa and correlation with mucosa-colonizing bacteria**. Co-occurrence network between ER stress-related genes in cluster 6 (**A**) and cluster 7 (**D**) and mucosa-colonizing bacteria (top 47). Co-occurrence network was constructed based on Spearman rank correlation coefficients (∣Spearman Coef∣ ≥ 0.5, *P* < 0.05). Lines connecting different nodes indicate positive (green) or negative (red) correlation between bacteria and genes, and line diameter is positively related to correlation value. All bacteria were named to genus level unless otherwise noted in brackets. Correlation network of ER stress-related genes in cluster 6 (**B**) and cluster 7 (**E**). Circle area is positively correlated with number of connected genes. Correlation network was constructed based on Spearman rank correlation coefficients (∣Spearman Coef∣ ≥ 0.8, *P* < 0.05). Multiple testing correction: BH. Interaction network of 15 ER stress-related genes in cluster 6 (**C**) and cluster 7 (**F**). Circle or equilateral triangle areas are positively correlated with number of connected genes. Interaction between circle-labeled genes with others has been reported. Interaction between equilateral triangle-labeled genes with others was predicted based on primary structure of gene-coding proteins. Control population: n = 6; UC patient: n = 12; CD patient: n = 5.


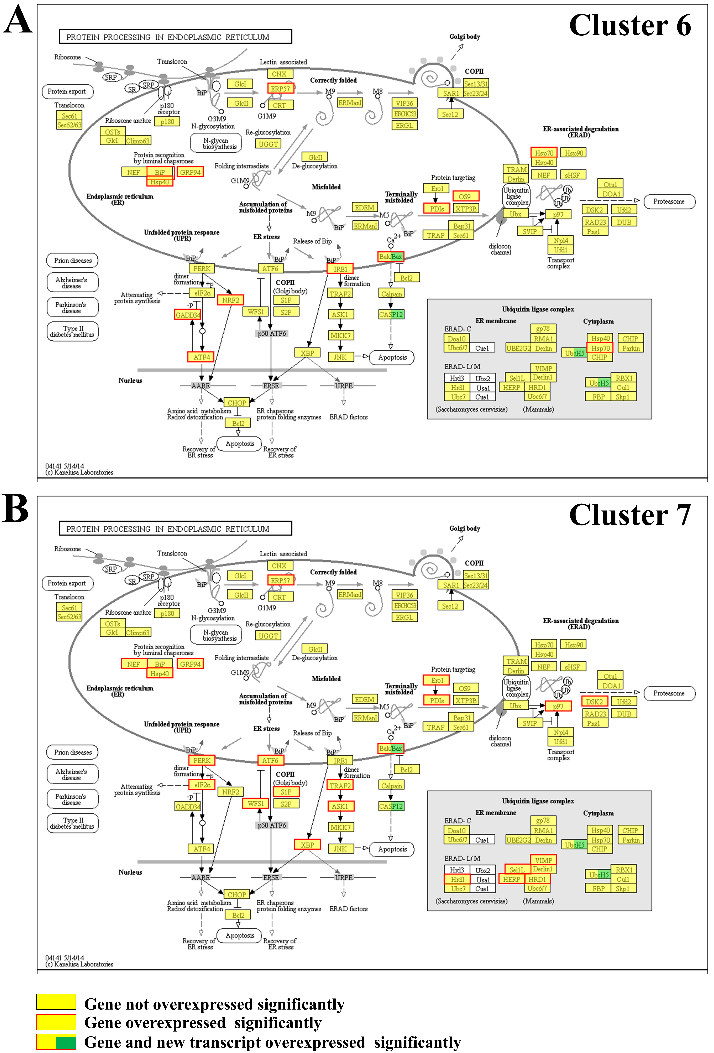


**Figure S11. KEGG ER stress enrichment analysis of cluster 6 (A), and cluster 7 (B).** Multiple testing correction: BH. Control population: n = 6; UC patient: n = 12; CD patient: n = 5.


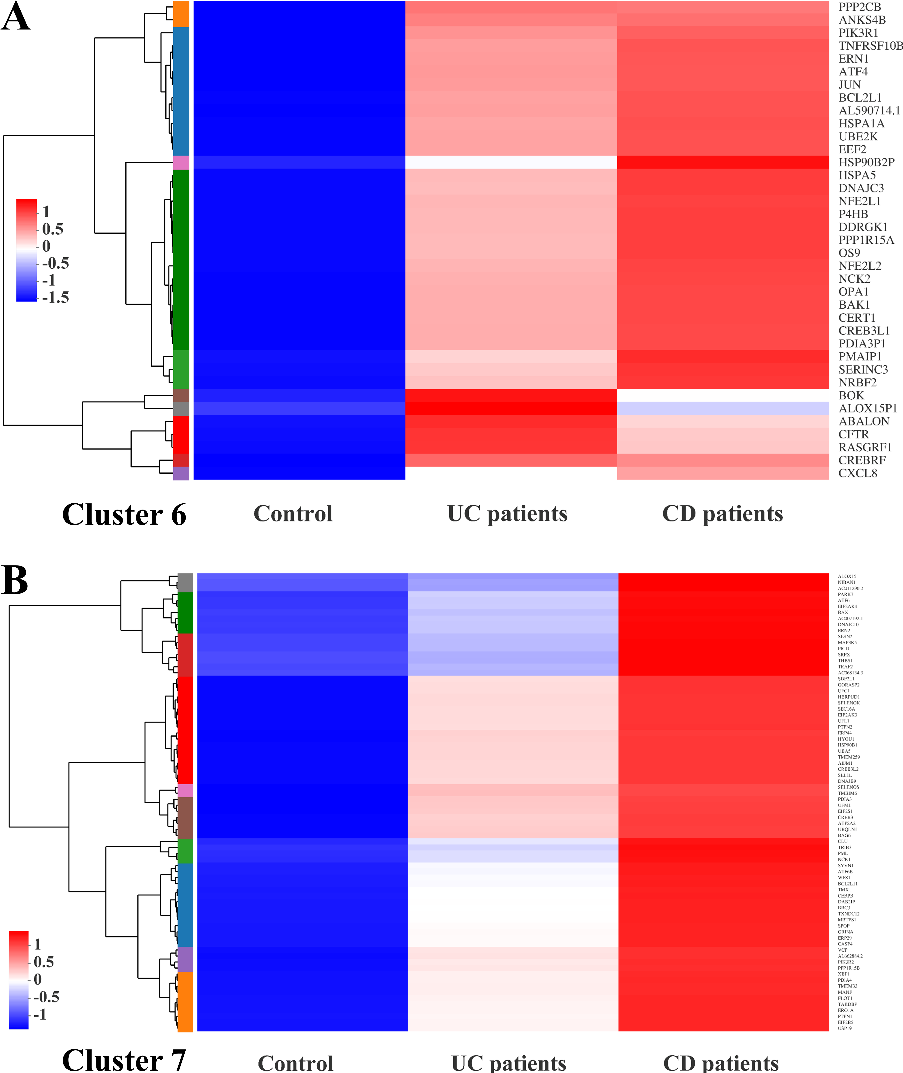


**Figure S12. Gene expression heatmaps of ER stress-related genes in cluster 6 (A) and cluster 7 (B) in intestinal mucosa of IBD patients.** Control population: n = 6; UC patient: n = 12; CD patient: n = 5.


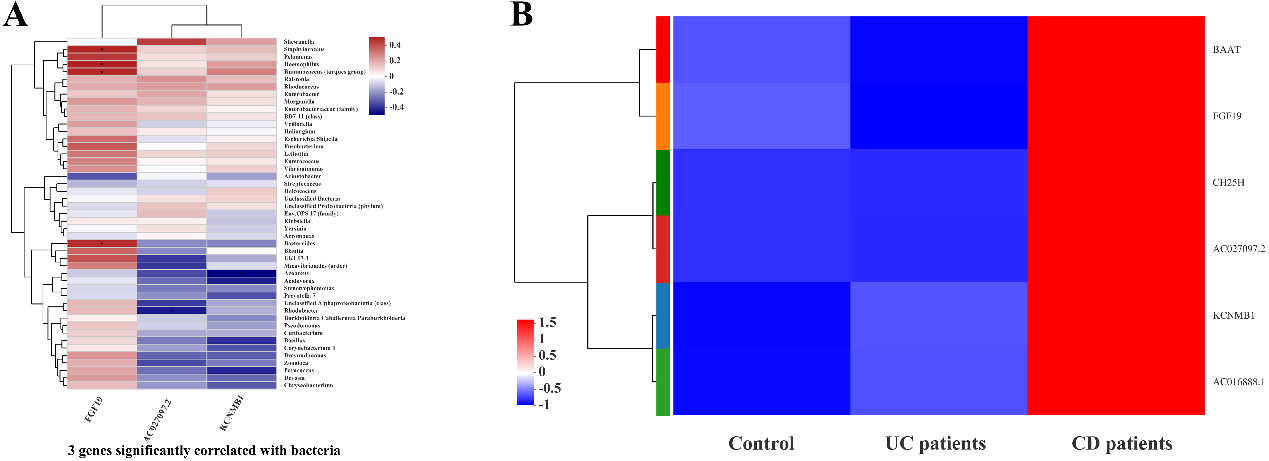


**Figure S13. (A) Spearman correlation heatmap shows three bile-related genes in cluster 4 were significantly correlated with mucosa-colonizing bacteria (top 47).** (**B**) Gene expression heatmap of bile acid production-related genes in cluster 4. All bacteria were named to genus level unless otherwise noted in brackets. * 0.01 < *P* ≤ 0.05, ** 0.001 < *P* ≤ 0.01, *** *P* ≤ 0.001. Control population: n = 6; UC patient: n = 12; CD patient: n = 5.


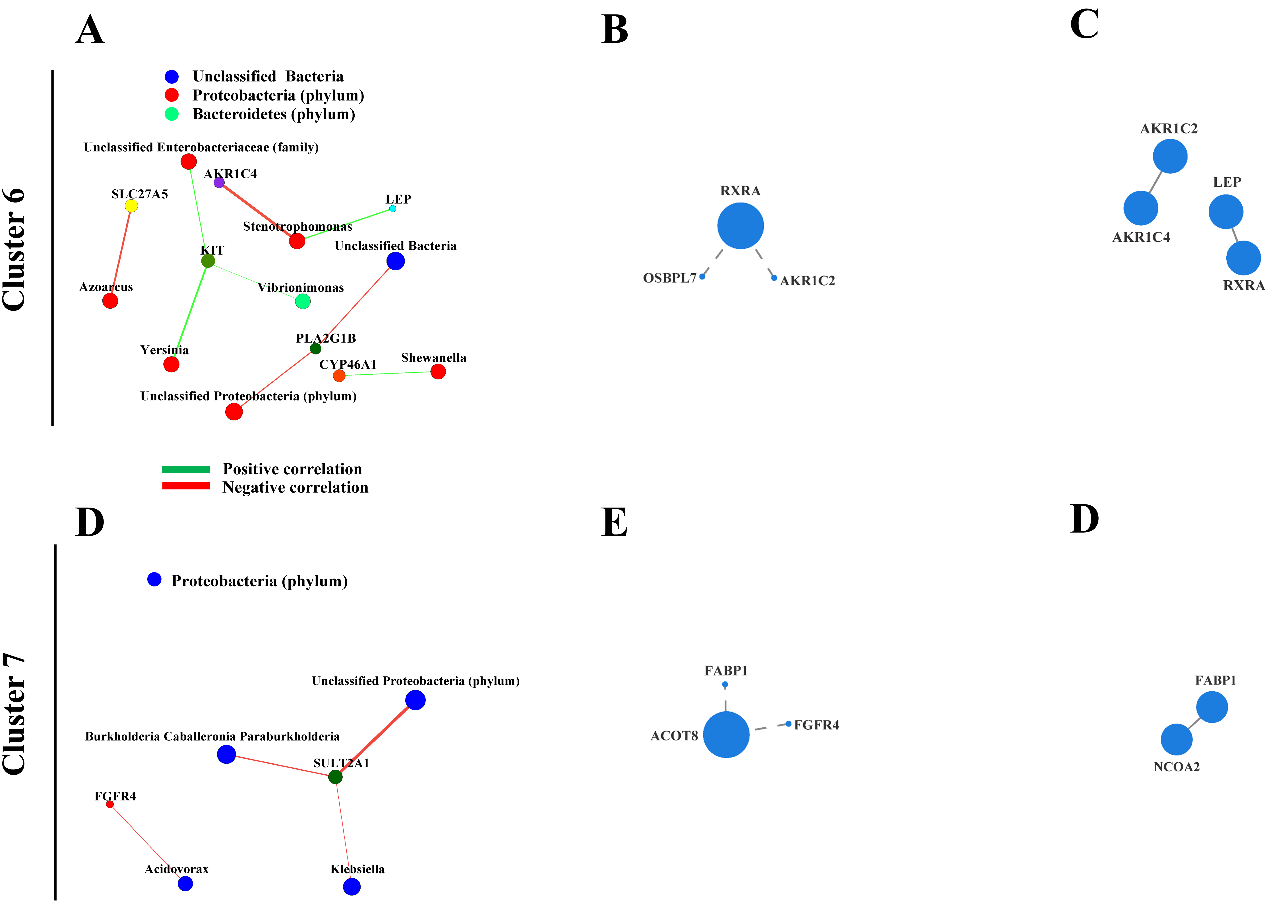


**Figure S14. Bile acid production** **network in intestinal mucosa and correlation with mucosa-colonizing bacteria**. Co-occurrence network between bile acid production-related genes in cluster 6 (**A**) and cluster 7 (**D**) and mucosa-colonizing bacteria (top 47). Co-occurrence network was constructed based on Spearman rank correlation coefficients (∣Spearman Coef∣ ≥ 0.5, *P* < 0.05). Lines connecting different nodes indicate positive (green) or negative (red) correlation between bacteria and genes, and line diameter is positively related to correlation value. All bacteria were named to genus level unless otherwise noted in brackets. Correlation network of bile acid production-related genes in cluster 6 (**B**) and cluster 7 (**E**). Circle area is positively correlated with number of connected genes. Correlation network was constructed based on Spearman rank correlation coefficients (∣Spearman Coef∣ ≥ 0.8, *P* < 0.05). Multiple testing correction: BH. Interaction network of bile acid production-related genes in cluster 6 (**C**) and cluster 7 (**F**). Circle or equilateral triangle areas are positively correlated with number of connected genes. Interaction between circle-labeled genes with others has been reported. Interaction between equilateral triangle-labeled genes with others was predicted based on primary structure of gene-coding proteins. Control population: n = 6; UC patient: n = 12; CD patient: n = 5.


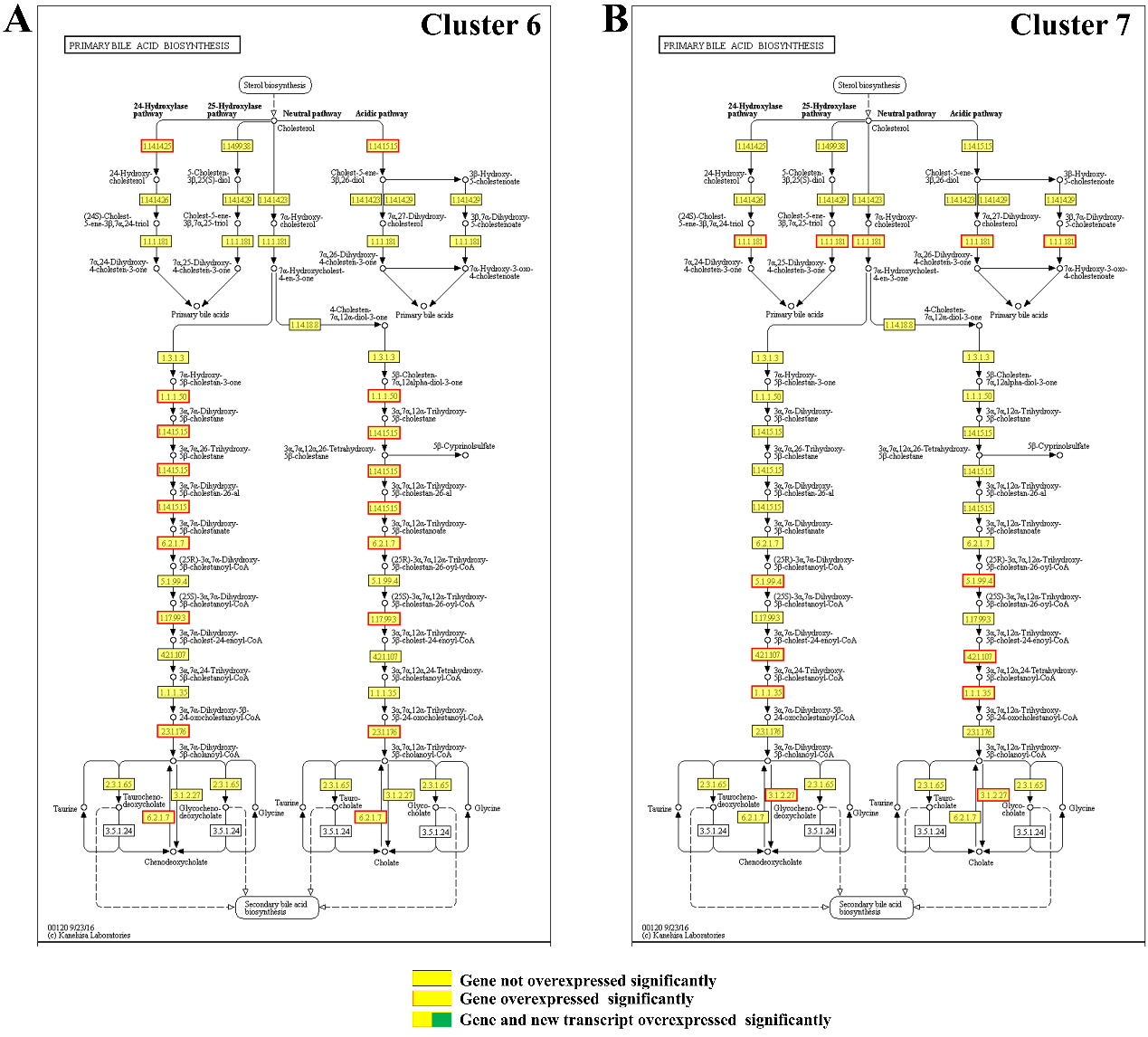


**Figure S15. KEGG functional enrichment analysis of bile acid biosynthesis in cluster 6 (A) and cluster 7 (B).** Multiple testing correction: BH. Control population: n = 6; UC patient: n = 12; CD patient: n = 5.


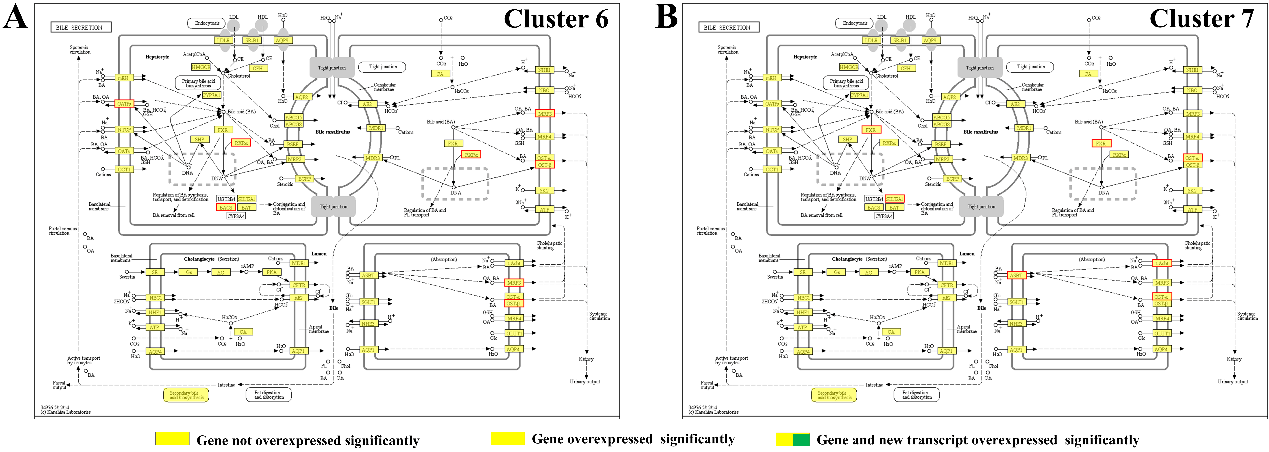


**Figure S16. KEGG functional enrichment analysis of bile acid secretion in cluster 6 (A) and cluster 7 (B).** Multiple testing correction: BH. Control population: n = 6; UC patient: n = 12; CD patient: n = 5.


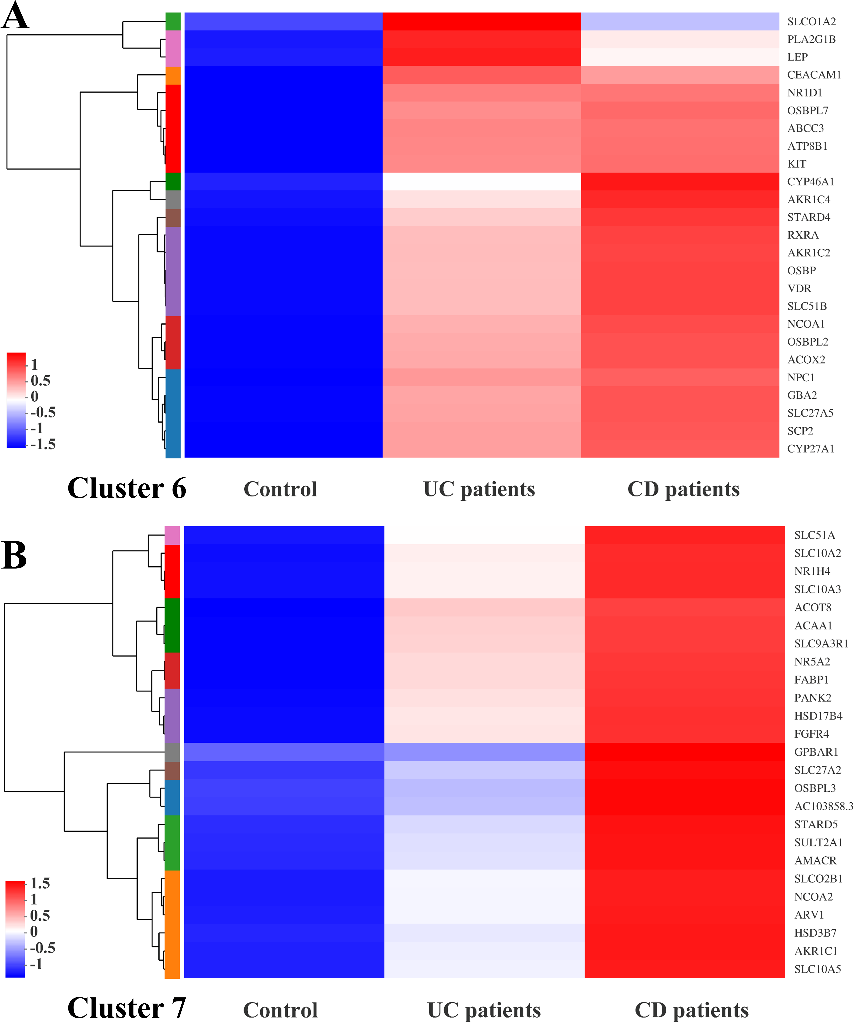


**Figure S17. Expression heatmaps of bile acid production-related genes in cluster 6 (A) and cluster 7 (B) in intestinal mucosa of IBD patients.** Control population: n = 6; UC patient: n = 12; CD patient: n = 5.

**Table S1**. Basic characteristics of 23 IBD patients selected for transcriptome analysis.

| **Index** | **Control**  **(n=6)** | **CD**  **(n = 5)** | **UC**  **(n = 12)** |
| --- | --- | --- | --- |
| Gender (male : female) | 3:3 | 3:2 | 7:5 |
| Age (mean ± SD) | 40.67±9.77 | 46.40±7.50 | 45.58±13.62 |
| Smoking (yes : no) | 0:6 | 0:5 | 2:10 |
| Alcoholic drinking (yes : no) | 1:5 | 0:5 | 5:7 |
| Antibiotic use  (within one months) | No | No | No |
| Occupation |  |  |  |
| Farmer | 0 | 0 | 4 |
| Factory worker | 4 | 4 | 4 |
| Office staff | 1 | 0 | 3 |
| Others | 1 | 1 | 1 |
| Chief complaint |  |  |  |
| Abdominal pain | 1 | 3 | 5 |
| Diarrhea | 0 | 2 | 3 |
| Blood in stools | 5 | 0 | 4 |
